# Subcellular protein turnover in human neural progenitor cells revealed by correlative electron microscopy and nanoscale secondary ion mass spectrometry imaging†

**DOI:** 10.1039/d3sc05629e

**Published:** 2024-01-29

**Authors:** Alicia A. Lork, Stefania Rabasco, Carl Ernst, André du Toit, Silvio O. Rizzoli, Nhu T. N. Phan

**Affiliations:** a Department of Chemistry and Molecular Biology University of Gothenburg SE-412 96 Gothenburg Sweden nhu.phan@chem.gu.se; b Human Genetics, McGill University H4H1R3 Montreal Canada; c Department of Neuro- and Sensory Physiology, University Medical Center Göttingen, Center for Biostructural Imaging of Neurodegeneration Göttingen Germany

## Abstract

Protein turnover is a critical process for accurate cellular function, in which damaged proteins in the cells are gradually replaced with newly synthesized ones. Many previous studies on cellular protein turnover have used stable isotopic labelling by amino acids in cell culture (SILAC), followed by proteomic bulk analysis. However, this approach does not take into account the heterogeneity observed at the single-cell and subcellular levels. To address this, we investigated the protein turnover of neural progenitor cells at the subcellular resolution, using correlative TEM and NanoSIMS imaging, relying on a pulse-chase analysis of isotopically-labelled protein precusors. Cellular protein turnover was found significantly heterogenous across individual organelles, which indicates a possible relation between protein turnover and subcellular activity. In addition, different isotopically-labelled amino acids provided different turnover patterns, in spite of all being protein precursors, suggesting that they undergo distinct protein synthesis and metabolic pathways at the subcellular level.

## Abstract 2

Protein turnover is a critical process for accurate cellular function, in which damaged proteins in the cells are gradually replaced with newly synthesized ones.

## Graphical abstract

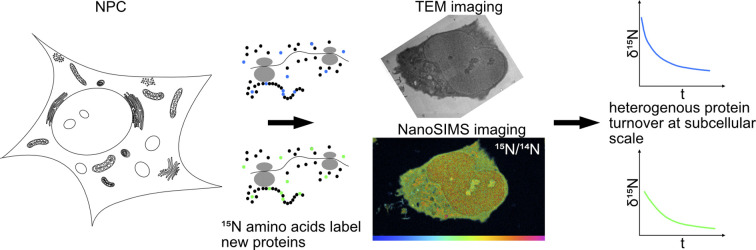

## Introduction

Protein turnover, the replacement of old or faulty proteins with newly synthesized ones, is a tightly regulated cellular process aimed at maintaining an intact proteome and ensuring proper functioning of cells. Disturbances in protein turnover can lead to accumulation of faulty proteins which can affect the viability of cells or lead to diseases.^1–3^ Protein turnover is dependent on transcription, translation, protein modifications and degradation and it has been shown to differ between different cell types^4^ and be influenced by the extracellular environment.^5^

Protein turnover is also a reflection of protein lifetime. The lifetimes of specific proteins have been determined in different mouse brain regions and the average lifetime was found to be around 10 days (most proteins had a lifetime from 3–13 days).^6^ DNA binding, signalling, and RNA binding proteins were found to have shorter lifetimes whereas histones, myelin and extracellular matrix proteins were found to be long lived. Synaptic long-lived proteins have been found in rodents in *in vitro* neuronal cultures and *in vivo*, and their lifetime was found to change when the activity of neurons was modulated pharmacologically.^7^ Nevertheless, the majority of synaptic proteins was found to have a shorter lifetime of 2–5 days in cultured rat cortical neurons.^8^

While providing valuable insight into the lifetimes of specific proteins, these data cannot provide insight into whether the protein's localization influences its lifetime. Yousefi *et al.* have shown with stimulated emission depletion (STED) microscopy that the localization of specific synaptic proteins correlates to their lifetimes.^9^ In addition, the protein turnover of differently active synapses in cultured hippocampal neurons was found to be highly heterogenous,^10^ highlighting the need to study the subcellular spatial organization of protein turnover, especially in highly polarized cells such as neurons and their precursors.

Nanoscale secondary ion mass spectrometry (NanoSIMS) imaging is a valuable tool in studying the subcellular distribution of analytes in mammalian cells.^10–15^ NanoSIMS has been used to study protein turnover in cells and tissues at a subcellular level for example in stress granules,^16^ stereocilia,^17^ synapses,^10^ and lysosomes.^18^ In NanoSIMS, a primary ion beam erodes a sample surface pixel by pixel, generating atomic and diatomic secondary ions from the sample surface. The ions of a selected polarity are then transferred into a magnetic mass analyser and further detected in parallel by up to 7 mass detectors. The results are ion images showing the spatial distribution of these detected ions across the sample surface. NanoSIMS offers high sensitivity of detection (detection limit of ppb–ppm), high mass resolution (*m*/Δ*m* ∼10 000), and especially, high spatial resolution (down to ∼50 nm),^19^ which makes it well-suited for studying the chemical composition at a subcellular level. NanoSIMS has been used with rare isotopic labelling to track protein turnover in cells and tissues.^16,20^ NanoSIMS is often combined with other imaging techniques such as fluorescence and/or electron microscopy to identify detailed morphological structures and obtain complementary information of sample properties.^10,21–23^ We have previously demonstrated correlative TEM and NanoSIMS imaging to assess the neurotransmitter content at the nanometer vesicles in cells.^13,14^

Protein turnover has been studied by other imaging techniques, especially fluorescence microscopy.^9,10^ Fluorescence imaging provides protein turnover information based on organelle specific proteins (for example, using TOM20 or TOM70 to represent mitochondria), and thus can only determine the turnover of these proteins but not the whole organelles. In addition, dysfunctional organelles, which may not express specific proteins, could be left out from the analysis.^22^ Our work using correlative TEM/NanoSIMS provides imaging of a collection of many organelles within single cells based on their morphological properties. This allows a wide range of comparisons of protein turnover across different types of organelles to obtain an insight into the molecular and functional relations between them.

In this study, we employed correlative transmission electron microscopy (TEM) and NanoSIMS imaging to study the subcellular protein turnover in neural progenitor cells (NPCs) derived from human induced pluripotent stem cells (hiPSC) using different isotopic amino acids as the protein precursors. Here, TEM imaging aided in the identification of specific subcellular structures, which are then correlated with the NanoSIMS images of the same cells to extract the corresponding turnover rates. We incubated NPCs for a determined period with one of the isotopic amino acids, ^15^N-containing leucine, proline, glycine, alanine or phenylalanine, which were uptaken by the cells and incorporated into newly synthesized proteins (pulse period). We then chemically fixed the cells at different time-points, following a 6 h to 96 h chase period after the amino acid incubation. By correlating TEM and NanoSIMS imaging, we could localize the incorporated ^15^N-containing amino acids in different organelles within single cells at various chase times. We found that protein turnover in different organelles was significantly different, and that different isotopic amino acids showed different apparent levels of protein turnover within the cells. In this study, we used TEM and NanoSIMS imaging as a valuable tool to investigate the subcellular maps of chemical composition and protein turnover in human neural progenitor cells. This is applicable to various biological questions related to protein turnover, such as protein turnover during the cell differentiation, development and degradation, protein homeostasis in cell and organismal behaviours, protein turnover in aging and neurodegenerative diseases, and manipulating protein turnover for therapeutic applications. With this study, we introduce a groundwork for further investigation into the functional regulation of protein turnover in iPSC-derived cells at the single organelle level.

## Material and methods

### Neural progenitor cell (NPC) culture

Midbrain NPCs derived from human induced pluripotent stem cells (hiPSCs) were obtained from the Carl Ernst lab, McGill University, Montreal, Canada. The use of these human cells was approved by the Research Ethics Board of the Centre intégré universitaire de santé et de services sociaux de l'Ouest-de-l'Île-de-Montréal with the ethics approval code F9H-749 and the date of approval of May 13, 2022. NPCs were thawed rapidly at 37 °C after which they were transferred to 10 ml of warm NPC medium (STEMDiff Neural Progenitor Basal Medium, Catalog #05834 STEMCELL technologies, Canada). The cells were centrifuged immediately at 1460 rpm for 5 min and the cell pellet was subsequently resuspended in NPC medium with 200 ng ml^−1^ Sonic Hedgehog (genescript Cat# Z03067); NPCs were plated onto a poly-d-lysine (Cat# P7280, Sigma-Aldrich, Sweden) and Laminin (Cat# L2020, Sigma-Aldrich, Sweden) coated T25 flask (Nunc™ EasYFlask™, Fisher Scientific, Sweden) and kept in an incubator at 37 °C and 5% CO_2_. Medium was exchanged every two days and the cells were propagated when they reached confluency. For protein turnover experiments, NPCs were plated on poly-d-lysine and laminin coated glass bottom dishes (MatTek Life Sciences, Ashland, MA USA), and were incubated with 4 mM ^15^N-containing amino acids for 48 h, followed by an incubation with regular NPC medium for a pre-determined chase period. Afterward, the cells were fixed by warm 2.5% glutaraldehyde in 0.1 M PIPES buffer.

### Sample preparation for correlative TEM and NanoSIMS

**Fig. 1:**
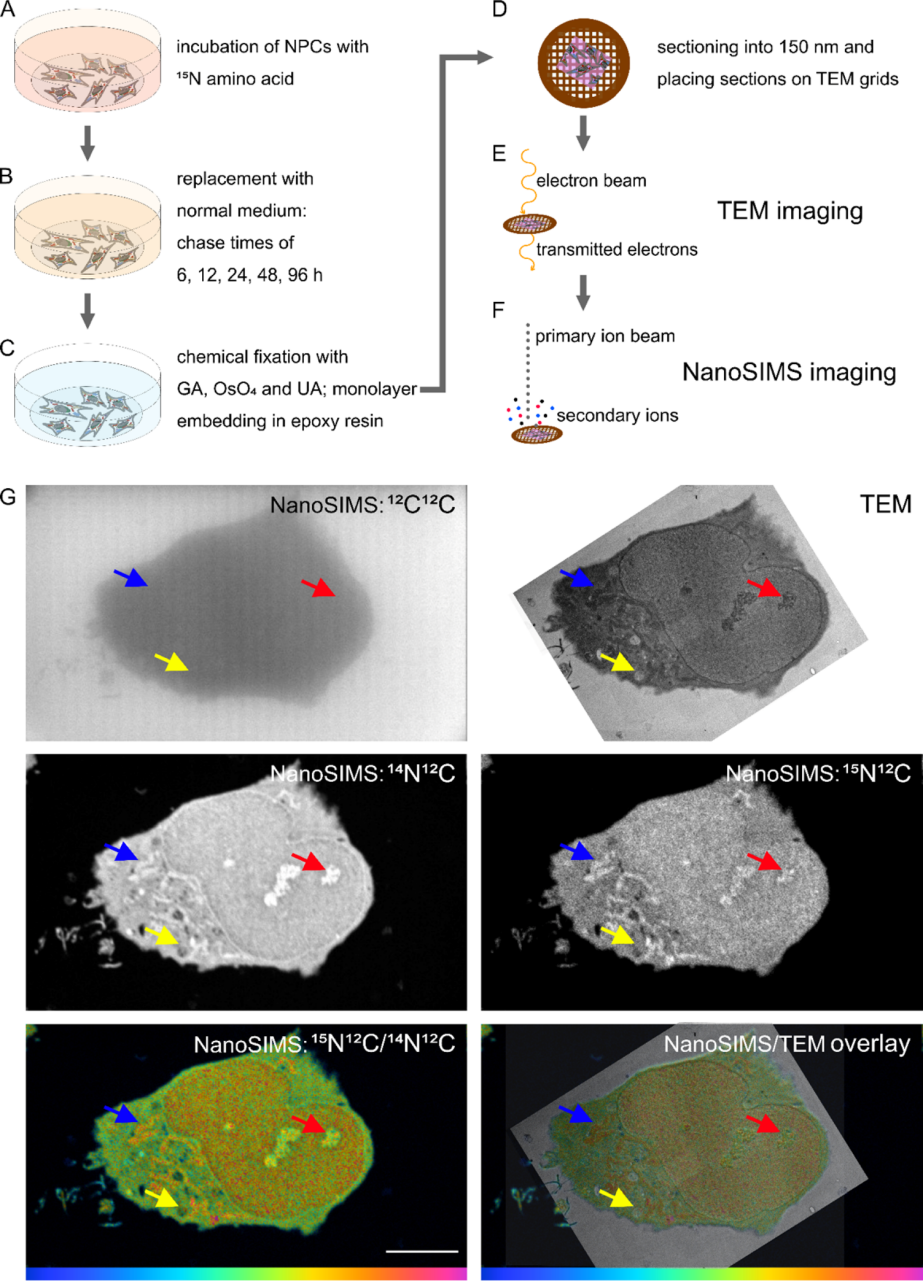
Schematic of sample preparation and correlative TEM and NanoSIMS imaging. (A) Samples are incubated with ^15^N-containing amino acid. (B) Cell medium with the labelled amino acid is replaced with regular cell medium and incubated for different time periods (chase time). (C) After the respective times, cells are fixed with Glutaraldehyde (GA), OsO_4_, and Uranyl Acetate (UA) and subsequently dehydrated and embedded in epoxy resin. (D) The resin block containing a monolayer of cells is then sectioned into 150 nm thick sections, which are placed on copper grids coated with Formvar. (E) Samples are imaged by TEM first, to obtain the ultrastructure of the cells. (F) The cell areas that were imaged with TEM are then imaged with NanoSIMS, to obtain their chemical composition. (G) Example NanoSIMS and TEM images of an NPC. TEM image, NanoSIMS ion images of ^12^C^12^C^−^, ^14^N^12^C^−^, ^15^N^12^C^−^, a hue saturation intensity (HSI) ratio image (^15^N^12^C^−^/^14^N^12^C^−^), and an overlay of TEM and HSI ratio images. NPCs were incubated with ^15^N-glycine for 48 h and underwent a chase period of 12 h. Arrows indicate different organelles which can be identified as mitochondria (blue), nucleolus (red), and lamellar inclusion (yellow). The colour scale represents the ^15^N^12^C^−^/^14^N^12^C^−^ ratio with blue at 0.0037 (natural abundance) and magenta at 0.04 (δ^15^N ∼ 1 × 10^4^‰). Scale bar 10 μm.

Samples were prepared for correlative TEM and NanoSIMS as previously described by Nguyen *et al.*^13^ Monolayer of cells embedded in resin were sectioned at 150 nm thickness. The workflow of sample preparation is presented in Fig. 1.

### TEM imaging

The sample sections were placed on copper finder grids with carbon support film (Electron Microscopy Sciences, FCF200F1-CU). TEM imaging was performed with a Thermo Scientific™ Talos L120C TEM microscope at the Centre for Cellular Imaging (Sahlgrenska Academy, University of Gothenburg, Sweden). The instrument was operated at 120 keV, and TEM images were taken at a magnification of 8500.

### NanoSIMS imaging

NanoSIMS imaging was performed with a NanoSIMS 50L (Cameca, France). Before the measurement, the samples were sputter coated with a thin film of gold to ensure good conductivity. A 16 keV Cs^+^ primary ion beam was used for implantations and image acquisitions. The same cells which had been imaged with TEM were located and implanted with a fluence of 3 × 10^16^ Cs^+^ per cm^2^. Image acquisition was performed with D1-4 (diaphragm width of 150 μm) and a primary ion beam current of 0.9–1 pA. Pixel size ranged from 45 to 82 nm with the FoV ranging from 23 to 42 μm. Dwell time was set at 5 ms per pixel. Each area was acquired with 5 consecutive image planes. Detectors were tuned to measure ^12^C_2_^−^, ^13^C^12^C^−^, ^12^C^14^N^−^, ^12^C^15^N^−^, ^31^P^−^, ^32^S^−^and ^34^S^−^. Mass resolving power of up to 10 296 was obtained at the mass 26.312 (^12^C^14^N^−^).

### Image analysis

TEM and NanoSIMS images were overlayed using Affinity Designer, where regions of interest (ROIs) of selected organelles were drawn on the TEM image for each cell. Binary images outlining the ROIs and matching the same pixel size of the corresponding NanoSIMS image were created and opened in ImageJ. The ROIs were automatically detected with the LabelstoROI plugin to ImageJ.^24^ Subsequently, NanoSIMS images were opened in the OpenMIMS plugin to ImageJ. Consecutive image planes were drift- and dead-time corrected, accumulated, and a ratio HSI image of ^12^C^15^N^−^/^12^C^14^N^−^ was generated. Isotopic ratios of ^12^C^15^N^−^/^12^C^14^N^−^ for designated ROIs were extracted, calculated into isotopic enrichment (δ^15^N) values (per mille, ‰), and then plotted with the software GraphPad Prism 9.3.1.

## Results and discussion

### Correlative TEM and NanoSIMS imaging as a tool for investigating subcellular turnover

To investigate subcellular protein turnover, a workflow of sample preparation and correlative TEM and NanoSIMS imaging is presented in Fig. 1. NPCs were incubated with isotopic (^15^N) amino acids for 48 h (Fig. 1A). This was followed by an incubation with regular cell medium for different periods allowing to track the uptake and subsequent clearance of the isotopic amino acid *via* newly synthesized proteins (Fig. 1B). Afterwards, the cells were chemically fixed with glutaraldehyde, then treated with osmium tetroxide and uranyl acetate, and embedded into epoxy resin as a monolayer to keep the cellular structure intact (Fig. 1C). The samples were subsequently cut into 150 nm sections and placed onto copper finder grids (Fig. 1D). TEM imaging was performed prior to NanoSIMS imaging (Fig. 1E and F) due to the former being non-destructive and the latter eroding the sample surface during the measurement. Secondary electron images can also be obtained with the NanoSIMS in parallel with the measurement of negative ions, however they only provide the information of morphology of the sample surface, and thus cannot be used to observe subcellular structures such as organelles beneath the sample surface (Fig. S1†). With the above-described workflow, TEM and NanoSIMS images of the same cell were obtained (Fig. 1G) and the isotopic ^15^N enrichment (δ^15^N) of specific organelles within the cell was extracted by overlaying the images from these imaging modalities.

Isotopic enrichment (δ^15^N) in per mille (‰) was calculated as the ratio of ^12^C^15^N^−^ over ^12^C^14^N^−^ above the natural atmospheric ratio (*r*_air_ = 3.7‰) using the following eqn (1):1
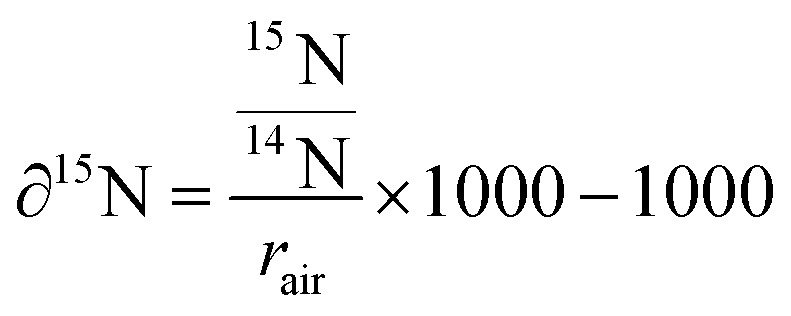
where ^15^N/^14^N is detected as ^12^C^15^N^−^/^12^C^14^N^−^. The Poisson uncertainty (eqn (2) in ESI†) of the measurements within the regions of interest (ROIs) typically ranged from 5–50‰ with the highest value of 120‰. Given the relatively high δ^15^N values measured in the cells (up to 22 323‰), this is deemed as an acceptable uncertainty. For the smallest, least ^15^N-enriched ROIs where the uncertainty is expected to be the highest, the uncertainty of each measurement was plotted in Fig. S2.†

Protein turnover can be tracked *via*^15^N enrichment in the cells owing to the ability of NanoSIMS, given the proposed sample preparation, to mainly detect the bound ^15^N-amino acid, which is mostly *via* incorporation into proteins.^25,26^ Small metabolites containing ^15^N-amino acids are not sufficiently fixed by glutaraldehyde and therefore will be washed away during multiple washing steps of the sample preparation. To support the argument that the ^15^N enrichment is mainly attributed to proteins, not small metabolites, we treated NPCs with a cocktail of protein synthesis inhibitors (puromycin, cycloheximide, and geneticin) and with ^15^N-labelled amino acid (^15^N-leucine or ^15^N-glycine). The results showed that the cells treated with protein synthesis inhibitors reduced the ^15^N enrichment by over 90% compared to that in the cells without the inhibitor treatment (Fig. S3†). This supports the claim that proteins are the major attribution to ^15^N enrichment in the cells.

Using the same experimental procedure, we could also measure simultaneously other abundant bound isotopes, ^31^P^−^ and ^32^S^−^. We first looked at the isotopic distribution from endogenous molecules, ^31^P^−^ and ^32^S^−^, across the single NPCs. Both ^31^P^−^ and ^32^S^−^ signals were pooled from all conditions and normalized to the respective ^12^C_2_^−^ signal (Fig. S4†). Across most of the identified organelles, the signals of ^32^S^−^ and ^31^P^−^ were shown to be significantly different based on the one-way ANOVA tests (Kruskal Wallis, Dunn's multiple comparison) (Tables S1 and S2†). Mitochondria and centrosomes show increased levels of ^32^S^−^ compared to other cellular compartments, which is possibly explained by a high protein density in these organelles compared to other compartments. It is possible that methionine and cysteine, amino acids containing sulphur, are present in a high abundance in these organelles leading to a high content of ^32^S^−^. In addition, a high amount of iron sulphur clusters, which are involved in DNA metabolism and are needed for mediating electron transfer in mitochondria, can give rise to a high content of ^32^S.^27,28^ On the other hand, the highest level of ^31^P^−^ was observed in nucleoli, nucleus, and lamellar inclusions, most likely due to an elevated content of nucleic acids and phospholipids in these structures.

### Protein turnover is heterogeneous at individual organelles in NPCs

We examined the subcellular protein turnover of NPCs *via* the spatial distribution of ^15^N enrichment across the cell using precursor ^15^N-glycine. It can be endogenously synthesized (*e.g.*, from serine,^29,30^ threonine^31^ and choline^32^) but usually not at a sufficient level. Moreover, glycine is also a neurotransmitter which, upon binding to its receptor, allows chlorine ions to diffuse across the membrane.^33^

**Fig. 2:**
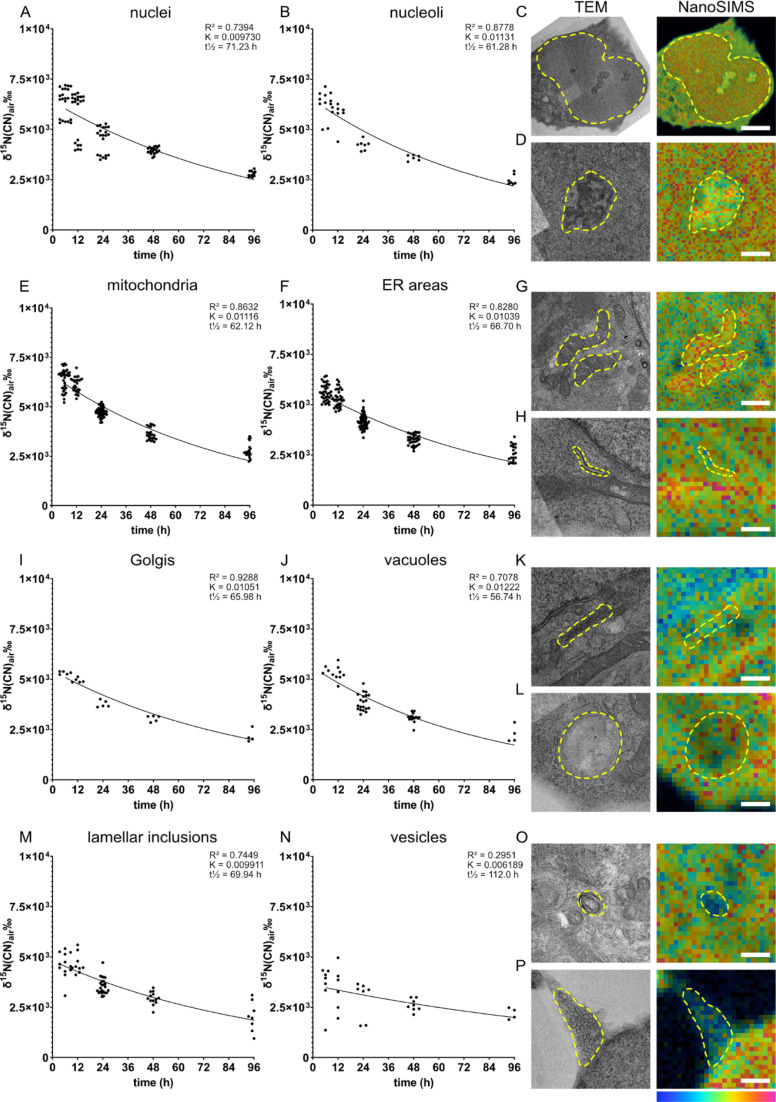
Protein turnover is different across different cellular compartments of single NPCs. NPCs were incubated with ^15^N-glycine for 48 h, and then with regular cell medium for different time periods (chase time: 6, 12, 24, 48, 96 h). The ^15^N turnover rate is plotted for each organelle and example correlative TEM and NanoSIMS images of the corresponding organelle are shown. (A&C) Nuclei, scale bar 5 μm in C; (B&D) Nucleoli, scale bar 1 μm in D. (E&G) Mitochondria, scale bar 1 μm in G; (F&H) ER areas, scale bar 500 nm in H. (I&K) Golgi, scale bar 500 nm in K; (J&L) vacuoles, scale bar 500 nm in L. (M&O) lamellar inclusions, scale bar 500 nm; (N&P) vesicles, scale bar 500 nm in P. A one-phase decay curve is fitted to each graph using GraphPad Prism software (constraints: Y0 > 0, plateau = 0, *K* > 0); *R*^2^ coefficient of determination, *K* rate constant, and *t*_1/2_ half-life. Example images are from cells incubated with ^15^N-glycine and 12 h chase time. Cellular compartments are indicated with a yellow dashed line. The colour scale (bottom right) represents the ^15^N^12^C^−^/^14^N^12^C^−^ ratio ranging from the natural abundance (0.0037, blue) to δ^15^N ∼1 × 10^4^‰ (0.04, magenta) for all images.

Eight different cellular compartments were identified on the TEM images, *i.e.*, nucleus, nucleolus, mitochondria, endoplasmic reticulum (ER), Golgi, vacuoles, lamellar inclusions, and vesicles (Fig. 2C, D, G, H, K, L, O and P, respectively), and their ^15^N enrichment was measured from the overlaid NanoSIMS images of the same cells. The ^15^N enrichment of these compartments was examined at five chase time-points, from 6 to 96 h (Fig. 2). We found that the ^15^N enrichment is heterogenous across the organelles of individual NPCs. The cells fixed after the 6 h chase time following amino acid incubation exhibit the highest ^15^N enrichments, which then decrease at the later chase times following an exponential decay trend. This is consistent with other previous studies on the decay fit of protein turnover.^9^^13^C_6_-lysine amino acid from food has been shown to rapidly equilibrate (within 24 hours) as the main source of intake for protein synthesis in mouse brain using a mathematical model and SILAC experiments.^6^ Therefore, with an isotopic incubation period of 48 h used in our study, it is expected that the ^15^N signal of the protein turnover will drop almost immediately from 0 h when the isotopic incubation is finished. At the 6 h time-point, the nuclei, nucleoli and mitochondria display the highest ^15^N enrichments at ∼ δ^15^N = 6.3 × 10^3^‰ (Fig. 2A, δ^15^N = 6.35 × 10^3^‰, B δ^15^N = 6.30 × 10^3^‰ and E, δ^15^N = 6.38 × 10^3^‰), whereas vesicles show the lowest level at δ^15^N = 3.6 × 10^3^‰ (Fig. 2N). Other organelles exhibit a δ^15^N ranging between 4.5 - 5.6 × 10^3^‰ (Fig. 2 F, I, J and M). With increasing chase periods, all organelles of NPCs showed a decrease in ^15^N enrichment, although at different rates, indicating that the ^15^N-labelled proteins of particular organelles are replaced gradually by newly synthesized unlabelled proteins. The high turnover of nucleoli can be related to their function in ribosome biogenesis, cell cycle regulation and gene expression.^34^

The high turnover rate of mitochondria could be explained by a high energy demand in NPCs, as mitochondria generate energy through the Krebs cycle, but also by the ability of mitochondria to mediate cell growth.^35^ Previous studies in *in vivo* mouse brains and neurons showed that a majority of mitochondrial proteins have relatively low turnover due to their long lifetime (>10 days).^3^ A few short-lived mitochondrial proteins have faster turnover and tend to be involved in fatty acid metabolism and peroxisome function. In our study, mitochondria showed a high rate of turnover over 96 h chase period (*K* = 0.01116), which is among the highest turnovered organelles such as nucleoli and vacuoles. The difference between our finding and the previous study could be due to the immature state of our NPCs and differences in protein lifetimes *in vitro* compared to *in vivo*. Recent evidence showed that mitochondria play an important role in NPCs in regulating neurogenesis processes and the metabolic programming required for cell fate decision.^36^ These regulating functions together with the classical role of mitochondria as an energy generator for cellular homeostasis could lead to their highly active function in NPCs. How actively the cellular compartments function, in turn, has been shown to closely relate to protein turnover, particularly a strong connection was found between single synaptic activity and protein turnover.^10^ Therefore, it is speculated that the high rate of protein turnover of mitochondria in NPCs is a result of an unusual high activity of the organelles for regulating cell fate and neurogenesis.

In contrast to mitochondria, vesicles exhibited the lowest ^15^N enrichment level and the lowest rate of protein turnover. Vesicles were shown to release around 200 times before being inactivated,^15^ and immature NPCs are expected to undergo far fewer neuronal secretion events compared to mature neurons, indicating a low need of protein synthesis and turnover of vesicular proteins.

It is noted that the level of ^12^C^14^N^−^ was shown significantly different across organelles. For example, a high abundance of ^12^C^14^N^−^ was found in mitochondria and nucleoli, whereas vesicles exhibited the lowest amount (Fig. S5 and Table S3†). ^12^C^14^N^−^ is a component of many biomolecules within cells such as nucleosides, phospholipids, and proteins. The finding here is consistent with the data obtained by Lange *et al.* who reported a heterogenous distribution of ^12^C^14^N^−^, as well as ^32^S^−^ and ^31^P^−^, across different organelles in HeLa cells.^22^ To test whether high ^15^N enrichment is due to high ^12^C^14^N^−^ content of the respective organelle, the relationship between ^12^C^15^N^−^ enrichment and ^12^C^14^N^−^ levels were illustrated in Fig. S6.† However, no consistent correlation between ^15^N enrichment and ^12^C^14^N^−^ levels was observed when looking at all the ROIs of each amino acid in each time-point. In addition, any correlation between ^15^N enrichment and ^12^C^14^N^−^ levels would be negligible when comparing ^15^N enrichment over time (turnover rate) between different organelles, therefore the differences in the protein turnover rate are due to the functional roles of the organelles and are not mere differences in the content of ^12^C^14^N^−^.

Despite being diluted by cell mitosis over the 96 h chase period, the level of ^15^N enrichment of all cells is still significantly above the natural abundance of 3.7‰ and the control cells (∼−38‰) which were incubated in regular cell medium without ^15^N-glycine (Fig. S7†). The dilution due to cell mitosis does not appear to be the dominating factor for the decrease in ^15^N enrichment as confirmed by an experiment with NPC-derived postmitotic cells, which were cultured with ^15^N-glycine in a differentiation cell medium inhibiting cell mitosis. The result showed a decreasing trend of ^15^N enrichment over a 96 h chase period similar to that obtained with the NPCs (Fig. S8†). This indicates that the decrease in ^15^N enrichment in the cells is mainly driven by the turnover of the cellular proteins, and not by cell division. The δ^15^N level after 96 h is also significantly above the δ^15^N in the resin surrounding the cells. The resin adjacent to the cells shows higher enrichment compared to that of resin areas further away from the cells, possibly due to the extracellular matrix components containing a certain ^15^N-labelled proteins having been secreted from the cells (Fig. S9†). The remaining elevated level of ^15^N over 96 h indicates a considerable proportion of proteins living longer than 96 h in the cells. In addition, it could simply be due to other metabolic pathways of ^15^N-glycine and its biosynthesis (*e.g.*, into serine),^37^ which further circulate ^15^N within the cellular system.

### Different protein precursors result in specific subcellular turnover patterns in NPCs

**Fig. 3:**
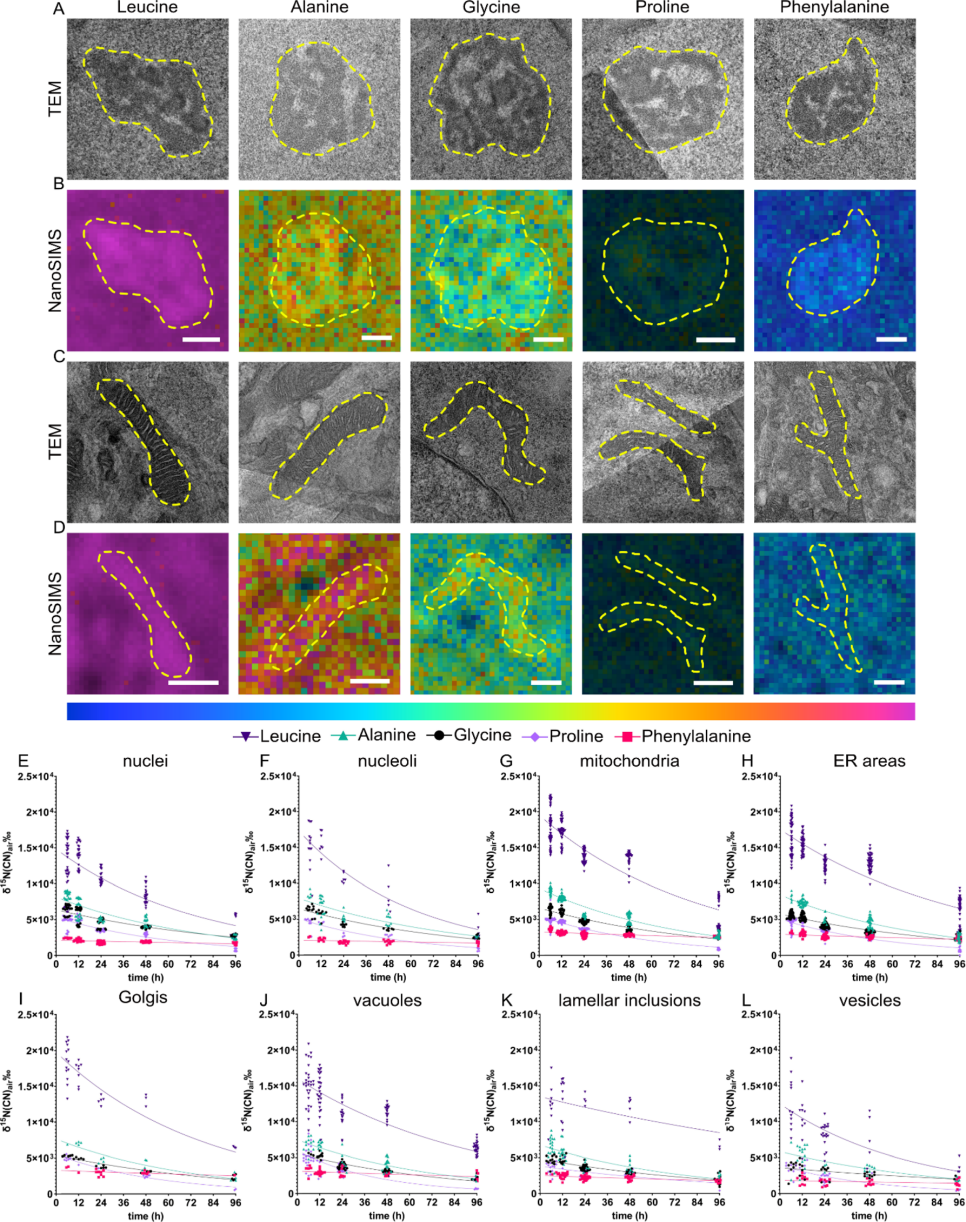
Different amino acids show distinguished protein turnover patterns at subcellular level. (A & C) Representative TEM images of (A) nucleoli and (C) mitochondria. (B & D) NanoSIMS ratio HSI image ^15^N^12^C^−^/^14^N^12^C^−^ for nucleoli and mitochondria with 12 h chase time after the incubation with ^15^N-leucine, ^15^N-alanine, ^15^N-glycine, ^15^N-proline, and ^15^N-phenylalanine (left to right). Cellular compartments are indicated with a dashed line. The colour scale (below D) represents the ^15^N^12^C^−^/^14^N^12^C^−^ ratio ranging from natural abundance level (0.0037, blue) to δ^15^N ∼1.2 × 10^4^‰ (0.05, magenta) for all NanoSIMS images. Scale bars are 500 nm. (E–L) ^15^N turnover rate plotted for the incubation with 5 different amino acids: ^15^N-leucine (dark purple) ^15^N-alanine (green), ^15^N-glycine (black), ^15^N-proline (light purple), and ^15^N-phenylalanine (pink) within different organelles. NanoSIMS measurements were obtained after ^15^N-amino acid incubation and a clearing period of 6, 12, 24, 48, 96 h. A one-phase decay curve was fitted to each dataset with GraphPad Prism 9.3.1 software.

Different amino acids are transported into the cell *via* uniporters, symporters and antiporters; for example glycine can be transported *via* symporter SNAT requiring Na^+^ as a cofactor whereas leucine is up-taken *via* antiporter LAT1.^38^ Some amino acids such as phenylalanine and leucine are termed “essential” as they cannot be synthesized by the body and therefore need to be supplied exogenously. The different amino acids participate in different functions of a protein due to their different charges, polarity, or hydrophobicity. In addition, amino acids can be transformed into other amino acids,^37^ neurotransmitters (*e.g.*, tyrosine, phenylalanine)^39^ and other metabolites.^40^ Therefore, different amino acids are likely to contribute differently to the overall protein homeostasis within the cells. We compared the subcellular protein turnover from five ^15^N containing amino acids, which differ in the size of the side chain, and being aliphatic or aromatic. Similar to the previous section, eight different cellular compartments were identified (Fig. 3A–D) and ^15^N enrichment of those was relatively quantified using correlative TEM and NanoSIMS imaging. We observed that the incorporation of these amino acids into the cells is largely different. NPCs incubated with ^15^N-leucine, one of the most frequently encoded amino acids, display the highest ^15^N enrichment, ranging up to 2.2 × 10^4^‰, compared to the other amino acids in all organelles, in a range of δ^15^N ∼2 × 10^3^–1 × 10^4^‰ (Fig. 3E–L). The lowest was found with ^15^N-phenylalanine which is incorporated into the nucleus and nucleoli at δ^15^N ∼2.5 × 10^3^‰. In addition, the enrichment in specific organelles is varied for particular isotopic amino acids. Interestingly, mitochondria were found to have the highest ^15^N abundance, whereas the lowest amount of ^15^N was found in vesicles, followed by lamellar inclusions, for all the five ^15^N containing amino acids. Golgi showed the highest ^15^N level in the cells incubated with ^15^N-leucine, and ^15^N-phenylalanine. On the other hand, nuclei and nucleoli showed the highest ^15^N level with ^15^N-glycine, but the lowest with ^15^N-phenylalanine. ER and vacuoles mostly exhibited a mid-range ^15^N enrichment.

There is a variation in the ^15^N enrichment within the analysed cells, especially the variation for ^15^N-leucine incubation is significantly larger than those for other amino acids, noticeably at the first and second chase time points (Fig. 3E–L, and S10†). The variation is possibly caused by the varied phases of the cell cycle in NPCs, *e.g.*, S-phase (DNA synthesis) or G1/G2-phase (cell growth), which could require different levels of protein synthesis and metabolism related to ^15^N-amino acids, hence leading to a biological variation among cells. Leucine showed the largest variation which could be due to its initial enrichment level being much higher than those of the others, thus a larger scale of variation is observed.

The clearing of ^15^N within these organelles was also different over the chase period of 6–96 h (Fig. 3E–L). The one-phase decay fits of the turnover across organelles for each ^15^N-amino acid are presented in Fig. S11–14.† Across all the examined organelles, ^15^N-leucine was enriched the most but did not exhibit the highest turnover, which was observed with ^15^N-proline (for example, the protein half-life *t*_1/2_ ∼ 59 h in mitochondria for leucine but *t*_1/2_ ∼ 42 h for proline, Fig. S11 and S12†). Interestingly, ^15^N-phenylalanine showed a significantly lower turnover rate compared to the other amino acids. The ^15^N enrichment detected in all organelles over 96 h chase time for ^15^N-phenylalanine was only slightly lower than the initial enrichment. On the other hand, ^15^N-alanine displayed the second highest ^15^N enrichment in all the organelles, after ^15^N-leucine, and followed by ^15^N-glycine and ^15^N-proline. The latter two were at an approximately similar enrichment level in most compartments, except for nucleoli and mitochondria. The turnover of these amino acids in different compartments, however, followed different rates. Among the five examined ^15^N containing amino acids, leucine (containing a large aliphatic chain) and phenylalanine (with an aromatic large side chain) show the largest significant difference in the ^15^N incorporation, with the aliphatic one showing the highest enrichment and the aromatic one showing the lowest. The other smaller aliphatic chained ^15^N precursors have an overall enrichment below that of aliphatic large-chained leucine.

Most of the amino acids often undergo various metabolic pathways besides being precursors for protein synthesis. Leucine has been known to promote protein synthesis *via* an activation of the mammalian target of rapamycin (mTOR) pathway, and to be involved in energy production by converting to α-ketoisocaproate (α-KIC) and β-hydroxy-β-methylbutyrate (HMB).^41^ The role of leucine in stimulating protein synthesis was studied in muscles of neonatal pigs,^42^ rats,^43^ and humans.^44,45^ These metabolic functions, especially the stimulation of protein synthesis, could be the cause for the high initial level of enrichment of ^15^N-leucine compared to the other precursors.

In contrast to leucine, ^15^N-phenylalanine presented the lowest ^15^N incorporation as well as the lowest turnover. This could be due to free ^15^N-phenylalanine being oxidized generating m-tyrosine, which can be incorporated into proteins and result in cellular stress.^46^ Cellular stress in turn could inhibit the overall protein turnover,^16^ resulting in a flatter curve of the turnover of ^15^N-phenylalanine across all the cell compartments. NPCs incubated with ^15^N-phenylalanine show high enrichment in mitochondria after 6 h chase time.

For all the examined ^15^N-containing amino acids, the ^15^N enrichment in the cells does not return to the natural abundance, or to the levels seen in the resin surrounding the cell, over 96 h chase period except for proline. It is possible that the ^15^N-amino acids are metabolized into other molecules, *e.g.*, the derived metabolites which remained in the cell for longer time or other amino acids being incorporated back into proteins. Another possibility is that a proportion of proteins living longer than 96 h remain in the cells. This elevation of ^15^N enrichment after 96 h was not pronounced in the cells incubated with ^15^N-proline. Proline metabolism is the main pathway for generating glutamate and glutamine *via* a conversion into pyrroline-5-carboxylate (P5C) under the catalysis of proline dehydrogenase.^47^ Glutamate and glutamine play both roles as amino acid and neurotransmitters in the brain and are involved in multiple metabolic pathways.^48,49^ Glutamate itself is the most abundant free amino acid and the major excitatory neurotransmitter in mammalian brain.^50^ These extended metabolic and signalling pathways could explain the low level of ^15^N enrichment after 96 h, therefore also a high rate of turnover of proline compared to the other precursors having similar initial enrichment as observed in our data.

It is noted that NanoSIMS measurement only detects small, fragmented ions, therefore the identity of the molecules associated with the ^15^N label cannot be identified. However, our data indicates that different protein precursors undergo specific protein synthesis and cellular metabolic pathways which result in distinguished subcellular patterns of protein turnover across the cell. Using NanoSIMS, it is possible to image and compare the differences in the protein turnover across different types of organelles, and even between the same organelles, within single cells (Fig. S15†), which has not been possible with pool analysis of single cell or subcellular fractionation by mass spectrometry proteomics.

### Molecular lifetime is influenced by subcellular localization and protein precursors

**Fig. 4:**
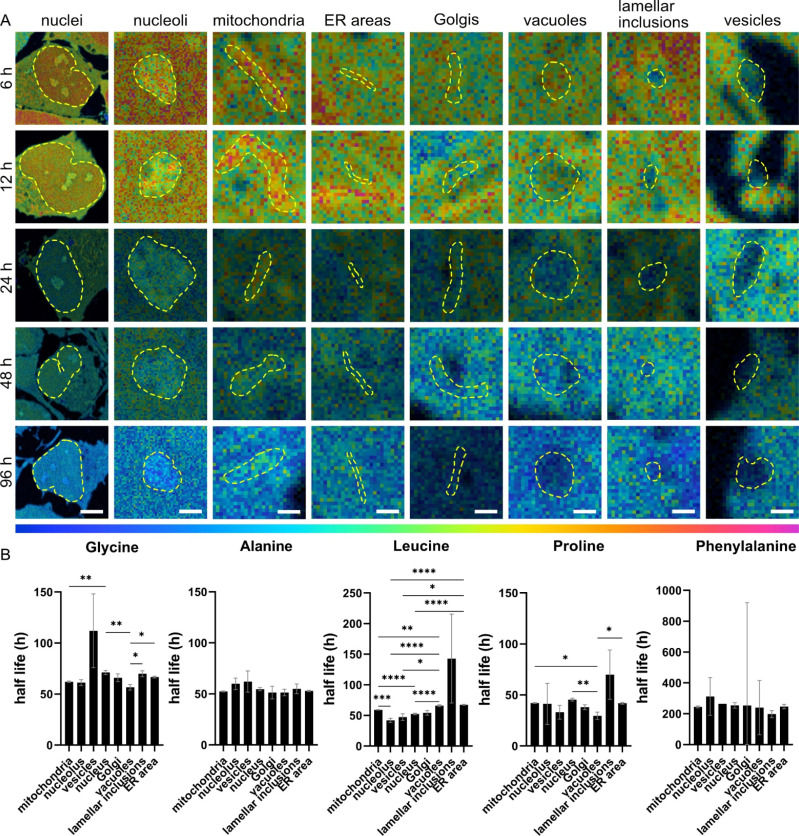
Protein lifetime within individual organelles. (A) NanoSIMS HSI ratio images of ^15^N^12^C^−^/^14^N^12^C^−^ for different organelles with different chase time of 6, 12, 24, 48, 96 h after ^15^N-glycine incubation. Cellular compartments (indicated with a dashed line) are identified by correlating with TEM images of the same cell areas. The colour scale (bottom) represents the ^15^N^12^C^−^/^14^N^12^C^−^ ratio ranging from natural abundance level (0.0037, blue) to δ^15^N ∼1.2 × 10^4^‰ (0.04, magenta) for all images. Scale bars for nuclei: 5 μm, for nucleoli 1 μm and 500 nm for all other organelles. (B) Protein half-lives (*t*_1/2_) within different subcellular compartments after incubation with different ^15^N-amino acids. SEMs for *t*_1/2_ were calculated from 95% confidence interval of the *t*_1/2_ of the one-phase decay fits. One-way ANOVA (Brown Forsythe and Welch) and subsequent Dunnett's multiple comparison test were performed, and significances are indicated above (*p < 0.05, **p < 0.01, ***p < 0.001, ****p < 0.0001).

We determined the half-lives of total proteins and molecules associated with ^15^N containing amino acids across different cellular compartments. The half-lives were found to be varied not only by compartments but also by ^15^N-amino acids (Fig. 4). First, NPCs incubated with ^15^N-proline showed the shortest half-lives in most of the organelles (in a range of 29–45 h), except for the lamellar inclusions (68 h), followed by ^15^N-leucine, ^15^N-alanine, ^15^N-glycine, and ^15^N-phenylalanine. This is as expected for ^15^N-proline due to its extensive metabolic and signalling pathways leading to a high rate of turnover, as explained earlier. Second, we observed that ^15^N-alanine has rather similar half-lives in all the organelles (within 10 h variation) with no statistically significant differences. This suggests that the subcellular turnover of ^15^N-alanine is homogenous and that its metabolism is rather consistent across organelles. Third, NPCs incubated with ^15^N-phenylalanine show extremely long half-lives of around 200 h or more. This could be due to cellular stress, as explained earlier, affecting cellular metabolism and turnover leading to a very slow clearance of the isotope. In turn, this affects the fit of the curve and the half-life calculation.

Protein half-lives were found significantly different across cellular organelles under different ^15^N-containing amino acids incubations. In Fig. 4B, the half-lives show the largest significant differences between the examined organelles following ^15^N-leucine incubation, except for lamellar inclusions due to their high variation. ^15^N-glycine and ^15^N-proline exhibit differences between a few organelles, such as mitochondria, nucleus and vacuoles, but at lower significance levels than ^15^N-leucine. Considering the protein half-lives within individual organelles from the four ^15^N-amino acids (^15^N-phenylalanine was excluded due to its cell stress effects, and thus the decay trend does not fit) (Fig. S16†), mitochondria show the largest difference between these precursors followed by ER, nucleus, and vacuoles. Interestingly, ^15^N-glycine and ^15^N-leucine result in rather similar protein half-lives in mitochondria, ER, and vacuoles, but significantly different ones in nucleus and nucleolus. On the other hand, vesicles and lamellar inclusions exhibit similar protein half-lives from all these precursors.

The data indicates that protein lifetime is influenced by subcellular localization, which is likely related to the biological functions of proteins within specific cellular structures. This is supported by both the *in vivo*, and *in vitro* data of other studies (Fig. S17†). Furthermore, the protein lifetime is also linked to the incorporating amino acids, which not only play as protein precursors but also as mediators in the crosswalks of multiple cellular metabolic pathways.

## Conclusions

We employed correlative TEM and NanoSIMS imaging to study subcellular protein turnover of human NPCs. The incorporation of isotopically labelled amino acids, as protein precursors, the protein turnover rates and the chemical composition were found significantly heterogenous at the organelle level. In addition, subcellular turnover patterns were distinguished between different precursors, possibly owing to their involvement in other cellular metabolic pathways. Furthermore, general protein lifetime was found to be influenced by subcellular localizations of proteins and by specific protein precursors. The novelty of our work is summarised in three main aspects. First, the finding about the heterogenous cellular protein turnover at the organelle level indicates a relation between protein turnover and subcellular activity. This possibly introduces a new paradigm for further discoveries in modulation of organelle turnover to influence cellular activity and processes. Second, the finding about the distinct subcellular turnover patterns for different amino acids provides a new perspective of subcellular protein turnover. This is particularly a very useful information in the field of protein turnover using SILAC method, where most studies use a single amino acid (primarily lysine) as a protein precursor, and thus could have a lysine bias. Third, the application of NanoSIMS to study protein turnover in human neural stem cells has never been done before at the subcellular level. Previous studies mostly focused on organelle specific proteins, but not a collection of organelles within single cells. This could be a groundwork for further studies in human stem cell biology to understand the subcellular regulation driving stem cell differentiation and stem cell fate.

We have demonstrated correlative TEM and NanoSIMS as a powerful integrative imaging tool to obtain complementary properties and composition of samples to investigate complex cellular processes such as molecular turnover. Applications of this method in highly polarized cells such as neurons and using detailed pulse-chase experiments with other metabolites to test the turnover of specific metabolic pathways will provide better insight into neuronal processes and brain biology. In this work, we compared the differences in turnover between different amino acids for each type of organelles in different groups of cells, meaning that each group of cells were incubated with one particular isotopically labelled amino acid. However, it would also be interesting to examine the turnover of multiple amino acids in the same cells for which labelling with multiple isotopic amino acids (*e.g.*^13^C^−^, ^15^N^−^ and ^18^O^−^) is needed. This can be considered for the future work.

## Data availability

Experimental data associated with the article are available upon request.

## Author contributions

Conceptualization: N. T. N. P, S. O. R., A. A. L.; funding acquisition: N. T. N. P.; resources: N. T. N. P., C. E.; methodology: N. T. N. P., A. A. L, C. E., S. O. R; sample preparation and data acquisition: A. A. L, S. R., A. T.; data analysis: A. A. L, S. R.; data interpretation: N. T. N. P., A. A. L, S. O. R.; visualization: A. A. L.; supervision: N. T. N. P.; writing – original draft: A. A. L, N. T. N. P. Writing – review and editing: all authors.

## Conflicts of interest

There are no conflicts to declare.

## Supplementary Material

SC-015-D3SC05629E-s001
