# Supplementary material for: Subcellular protein turnover in human neural progenitor cells revealed by correlative electron microscopy and nanoscale secondary ion mass spectrometry imaging
Source: Chem Sci. 2024 Jan 29;15(9):3311–22. doi: 10.1039/d3sc05629e (PMC10901485; doi:10.1039/d3sc05629e)
Supplement: SC-015-D3SC05629E-s001 [file SC-015-D3SC05629E-s001.pdf]

Electronic Supplementary Material (ESI) for Chemical Science.
This journal is © The Royal Society of Chemistry 2024

Subcellular Protein Turnover in Human Neural Progenitor Cells Revealed by Correlative Electron Microscopy and Nanoscale Secondary Ion Mass Spectrometry Imaging

Alicia A. Lork [a], Stefania Rabasco [a], Carl Ernst [b], André du Toit [a], Silvio O. Rizzoli [c] and Nhu T. N. Phan [a†]

[a.] *Department of Chemistry and Molecular Biology University of Gothenburg, SE-412 96 Gothenburg, Sweden*
[b.] *Human Genetics, McGill University, H4H1R3 Montreal, Canada*
[c.] *Department of Neuro- and Sensory Physiology, University Medical Center Göttingen; Center for Biostructural Imaging of Neurodegeneration, Göttingen, Germany*
† Corresponding author

Supplementary Information

Equation (2) to calculate the Poisson uncertainty for each ROI:

$$Poisson\ uncertainty\ (‰) = \frac{1000}{\sqrt{area\ (pixels) \cdot 12C15N\left(\frac{\frac{c}{s}}{pix}\right) \cdot dwelltime\ (s) \cdot number\ of\ cycles}}$$

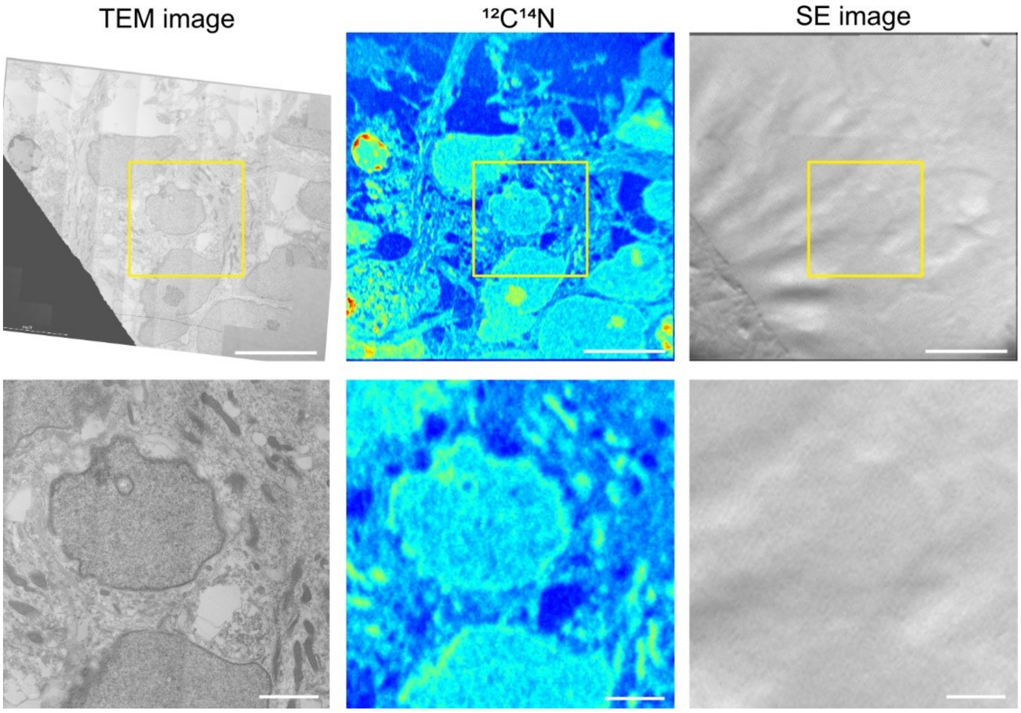

Figure S1: Images of cells imaged with correlative TEM and NanoSIMS. During NanoSIMS imaging in addition to detecting negatively charged ions (e.g. $^{12}C^{14}N^-$) secondary electrons (SE) were also detected. TEM is used to obtain the ultrastructure of the cells, $^{12}C^{14}N^-$ is detected within the cell and is heterogeneously distributed across the cell and the SE image shows the surface of the section

containing the cells. A zoom in of the area marked in yellow is shown below. Scale bars are 10 µm (upper row) and 5 µm (bottom row).

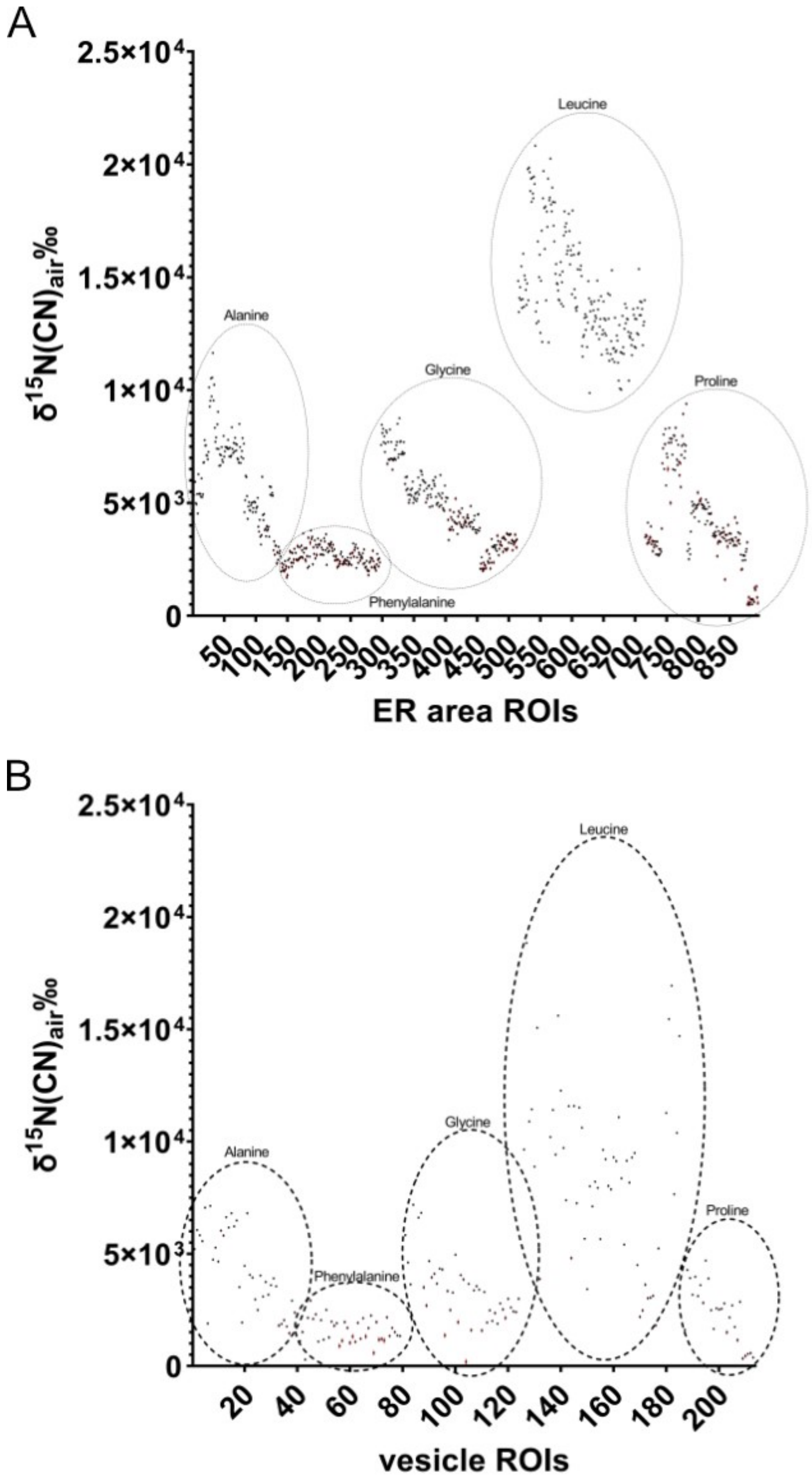

Figure S2: $\delta^{15}N$ plotted with the uncertainty of each measurement (error bar in red) across all the ROIs selected in different cells for different amino acids and chase times. (A) ROIs of Endoplasmic reticulum, (B) ROIs of vesicles.

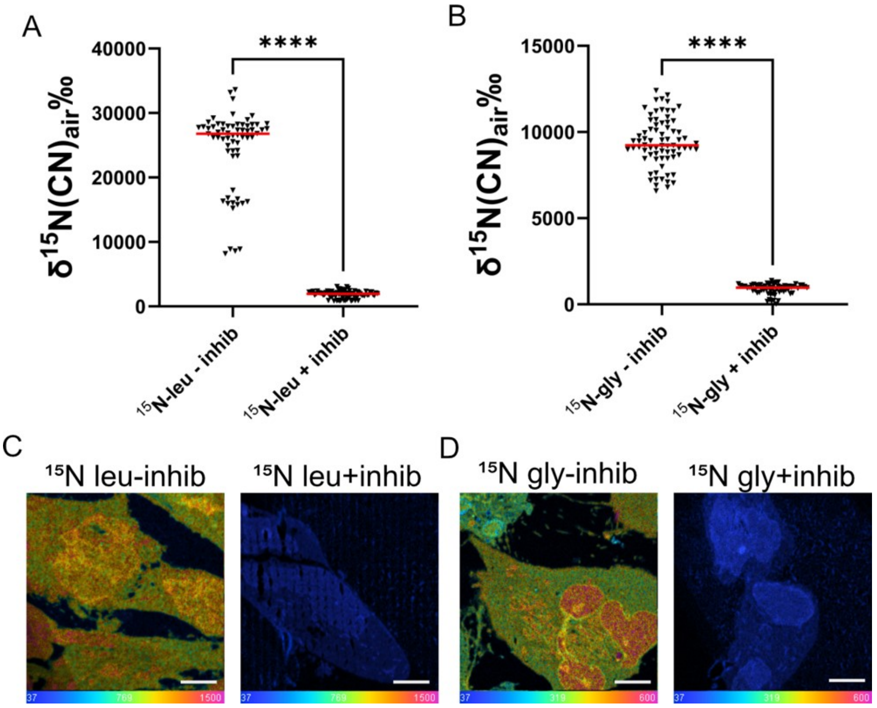

Figure S3: A-B) Comparison of the ^{15}N enrichment in the control NPCs (without protein synthesis inhibitors) and NPCs treated with inhibitors for protein synthesis (0.5 µg/mL Puromycin, 15 µg/mL Cyclohexamide, and 50 µg/mL geneticin, treatment with protein synthesis inhibitors started 4 h prior to isotopic amino acid incubation). A) Cells incubated with 4 mM ^{15}N-leucine for 40 h. B) Cells incubated with 4 mM ^{15}N-glycine for 40 h. The cells were then washed with PBS, chemically fixed, washed, embedded in resin, and cut into thin sections using the sample preparation mentioned in the Materials and Method section. Afterward, the cell slides were imaged with NanoSIMS to examine the ^{15}N enrichment present in the cells. C-D) $^{15}N/^{14}N$ NanoSIMS images of control NPCs and NPCs treated with inhibitors for protein synthesis. Two consecutive imaging planes (30x30 µm, 256x256 pixels) were acquired with a primary current of 2.7 pA with D1-4 aperture. C) Cells incubated with ^{15}N-leucine. D) Cells incubated with ^{15}N-glycine. Scale bars are 5 µm.

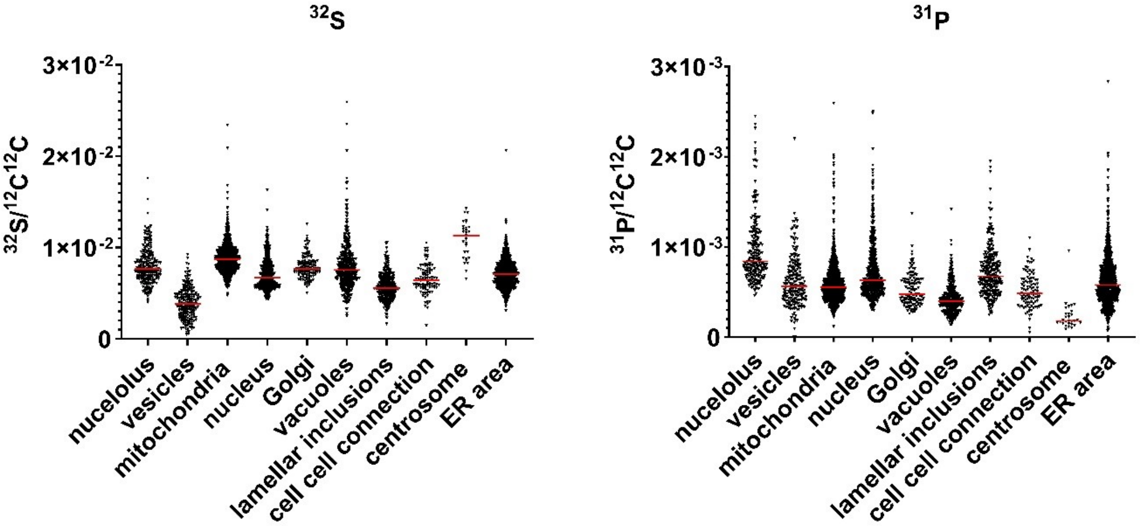

Figure S4: Levels of $^{32}S^-$ and $^{31}P^-$ in different organelles across the cells. Data was pooled from all incubation conditions and normalized to $^{12}C_2^-$. Significances are summarized in Table S1 and S2.

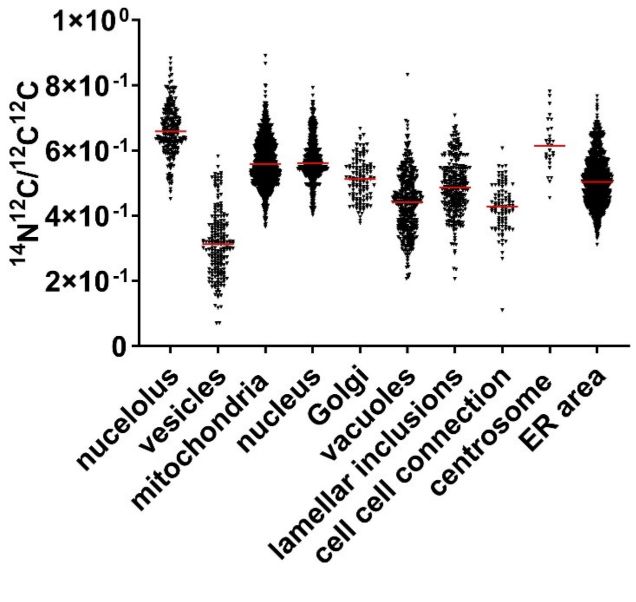

Figure S5: The $^{12}C^{14}N^-$ levels of different organelles were determined. Nucleolus and centrosomes have the highest $^{14}N^{12}C^-$ signal whereas vesicles have the lowest. The significances are summarized in Table S3.

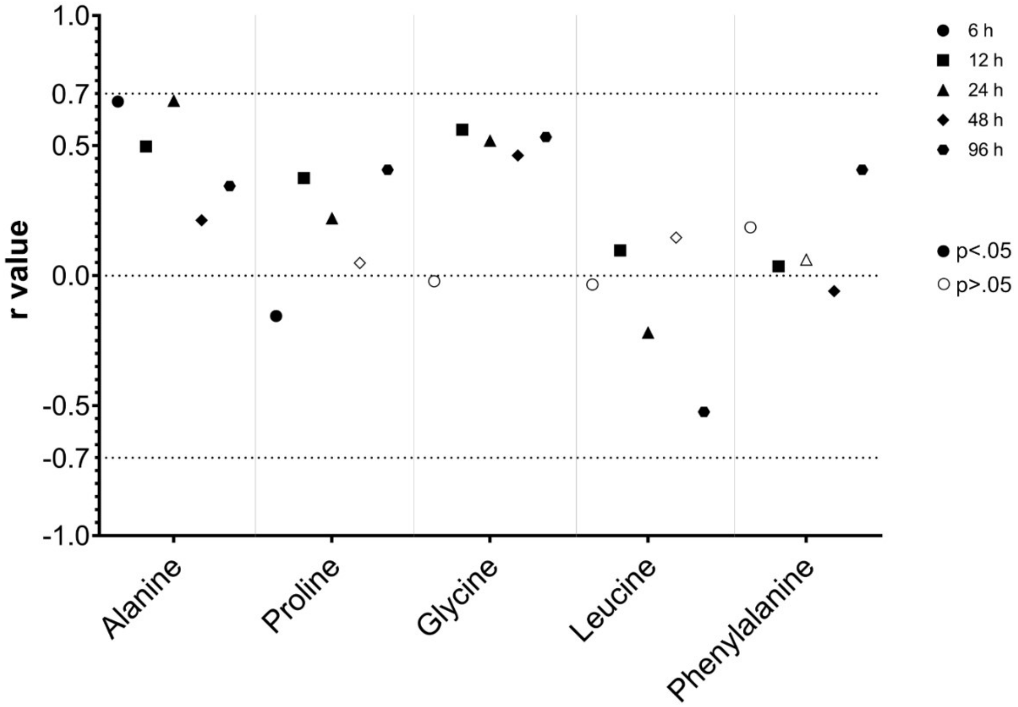

Figure S6: No consistent correlation is observed between $\delta^{15}N$ and $^{12}C^{14}N^-$ levels. The correlation coefficient value of each timepoint for each amino acid is plotted and the significance level of the correlation is indicated (Spearman correlation: black-filled symbols significant with $p < 0.05$, white-filled symbols not significant $p > 0.05$).

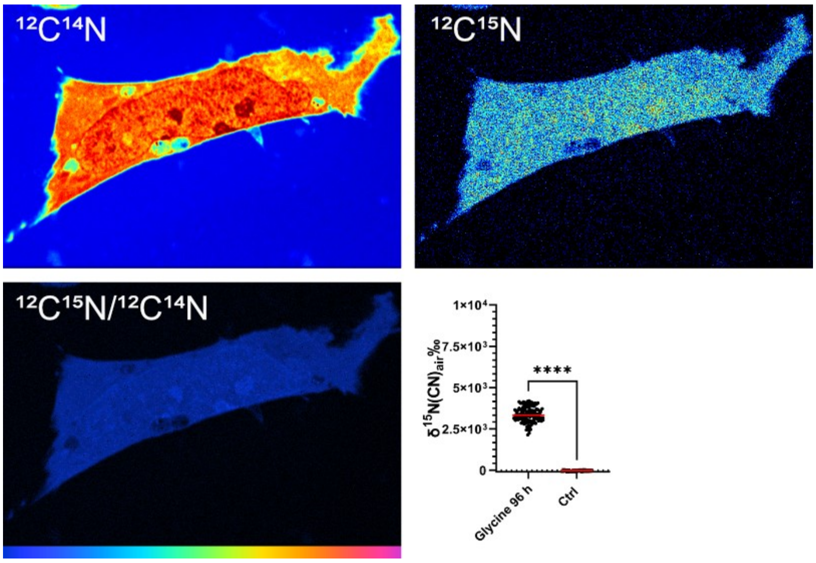

Figure S7: Ion images of $^{12}C^{14}N^-$, $^{12}C^{15}N^-$ and $^{12}C^{15}N^-/^{12}C^{14}N^-$ ratio HSI (color scale from blue: 0.0037 to magenta: 0.04) of a control cell incubated in regular cell medium without ^{15}N-amino acid. The $\delta^{15}N$ levels of the whole control cell and the cells incubated with ^{15}N-glycine followed by a 96 h chase period are significantly different (Mann-Whitney test; $p<.0001$). $\delta^{15}N$ levels are ~-38‰ for control whereas after 96 h of clearing time ^{15}N-glycine incubated cells are at ~3300‰

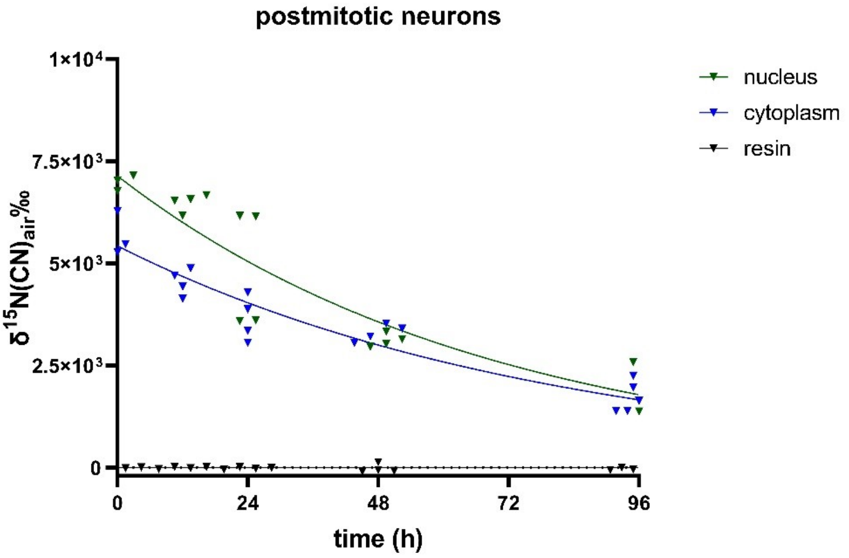

Figure S8: The ^{15}N enrichment over 96 h chase period of postmitotic neurons differentiated from midbrain NPCs incubated with 2 mM ^{15}N Glycine for 48 h on day 5 after the start of differentiation when the cell mitosis were efficiently inhibited.[1] For cell differentiation, NPCs were plated onto polyornithine and laminin coated Mattek dishes and medium was switched to DMEM/F12 supplemented with 2 % B27, 1 % N2, 20 ng/mL BDNF, 20 ng/mL GDNF, 200 nM Ascorbic Acid, 1 mM dibutyryl cAMP,1 µg/mL Laminin. Medium was exchanged partially (50:50) every two days. A one-phase decay curve is fitted with GraphPad Prism 9.3.1 software.

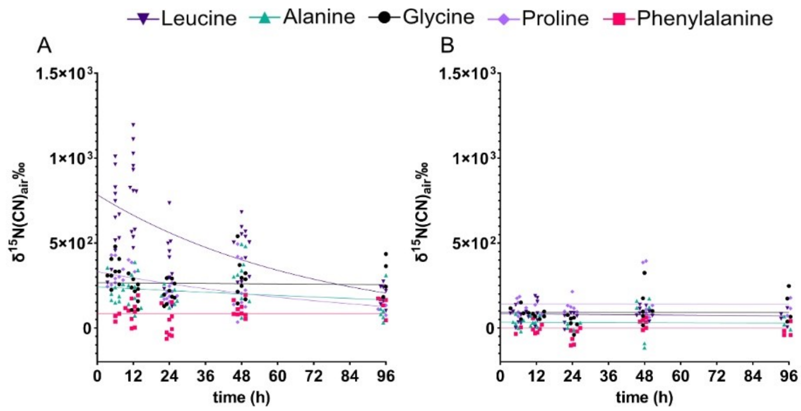

Figure S9: $\delta^{15}N$ of the embedding resin surrounding the NPCs incubated with ^{15}N-amino acids. $\delta^{15}N$ levels are different in the resin next to the cell compared to that further away from the cell. A) $\delta^{15}N$ of the resin close to the cell at different chase times. B) $\delta^{15}N$ of the resin further away from the cell at different chase times.

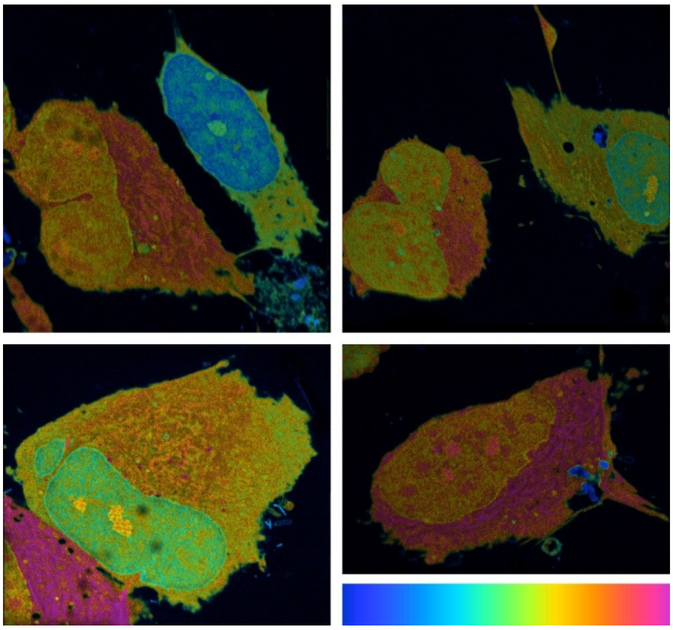

Figure S10: HSI images of ^{15}N Leucine incubated NPCs after 6 h chase time. Substantial biological variation is seen in the $\delta^{15}N$ between the cells. Color scale (blue to magenta) represents the ratio from 0.0037 (natural abundance) to 0.085 ($\delta^{15}N$~$2.2x10^4$ ‰).

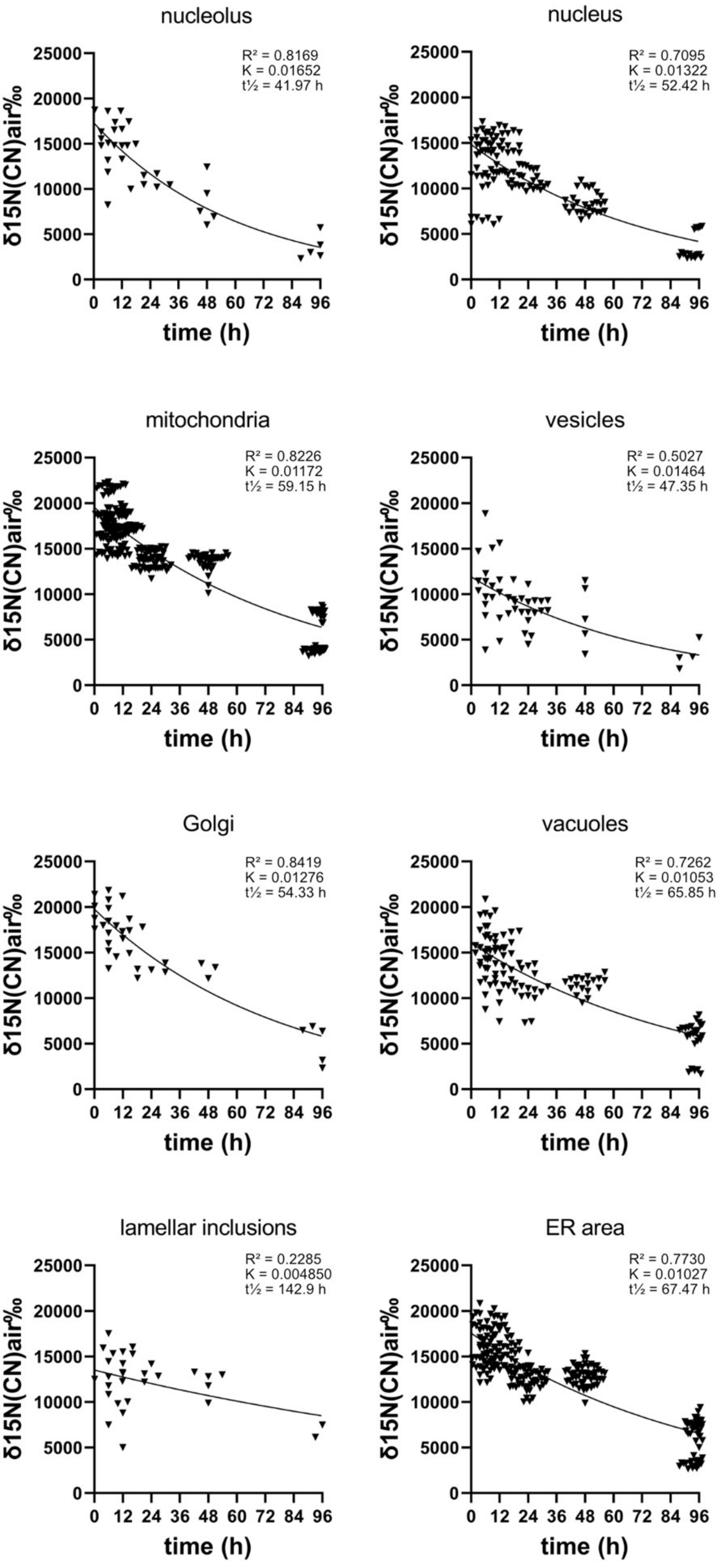

Figure S11: $\delta^{15}N$ over 96h chase time in ^{15}N-leucine incubated cells. A one-phase decay curve is fitted to each dataset with GraphPad Prism 9.3.1 software; R^2, rate constant K and $t_{1/2}$ are given for each curve.

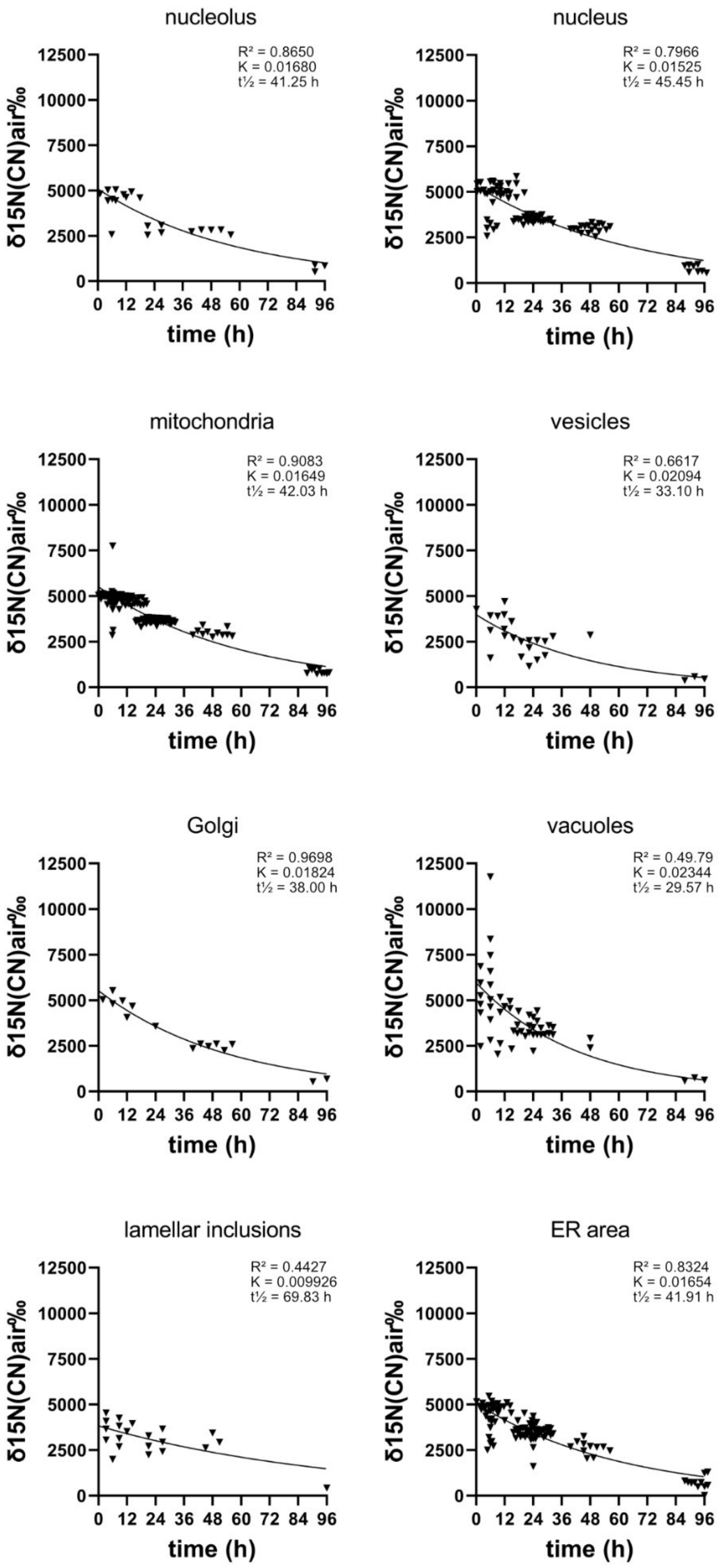

Figure S12: $\delta^{15}N$ over 96h chase time in ^{15}N-proline incubated cells. A one-phase decay curve is fitted to each dataset with GraphPad Prism 9.3.1 software; R^2, rate constant K and $t_{1/2}$ are given for each curve.

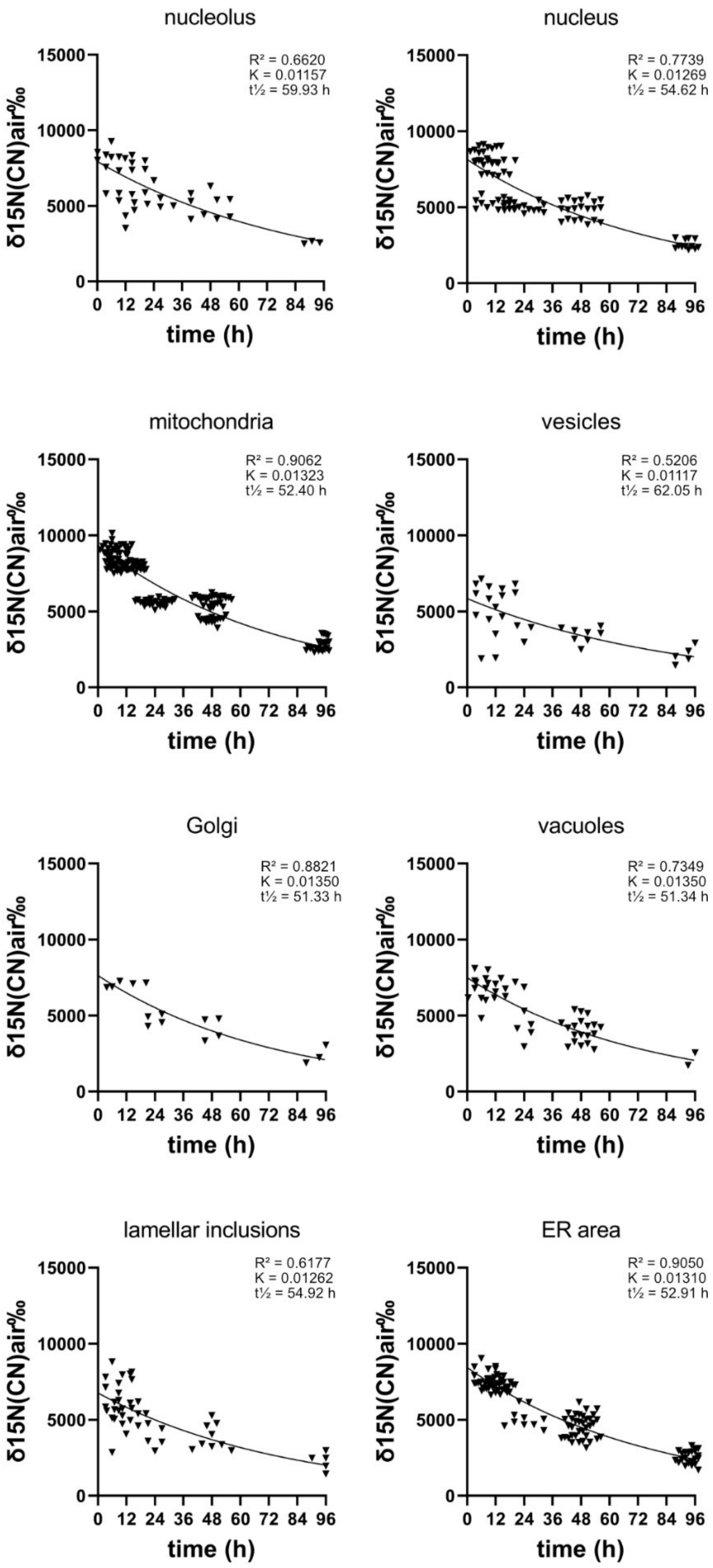

Figure S13: $\delta^{15}N$ over 96h chase time in ^{15}N-alanine incubated cells. A one-phase decay curve is fitted to each dataset with GraphPad Prism 9.3.1 software; R^2, rate constant K and $t_{1/2}$ are given for each curve.

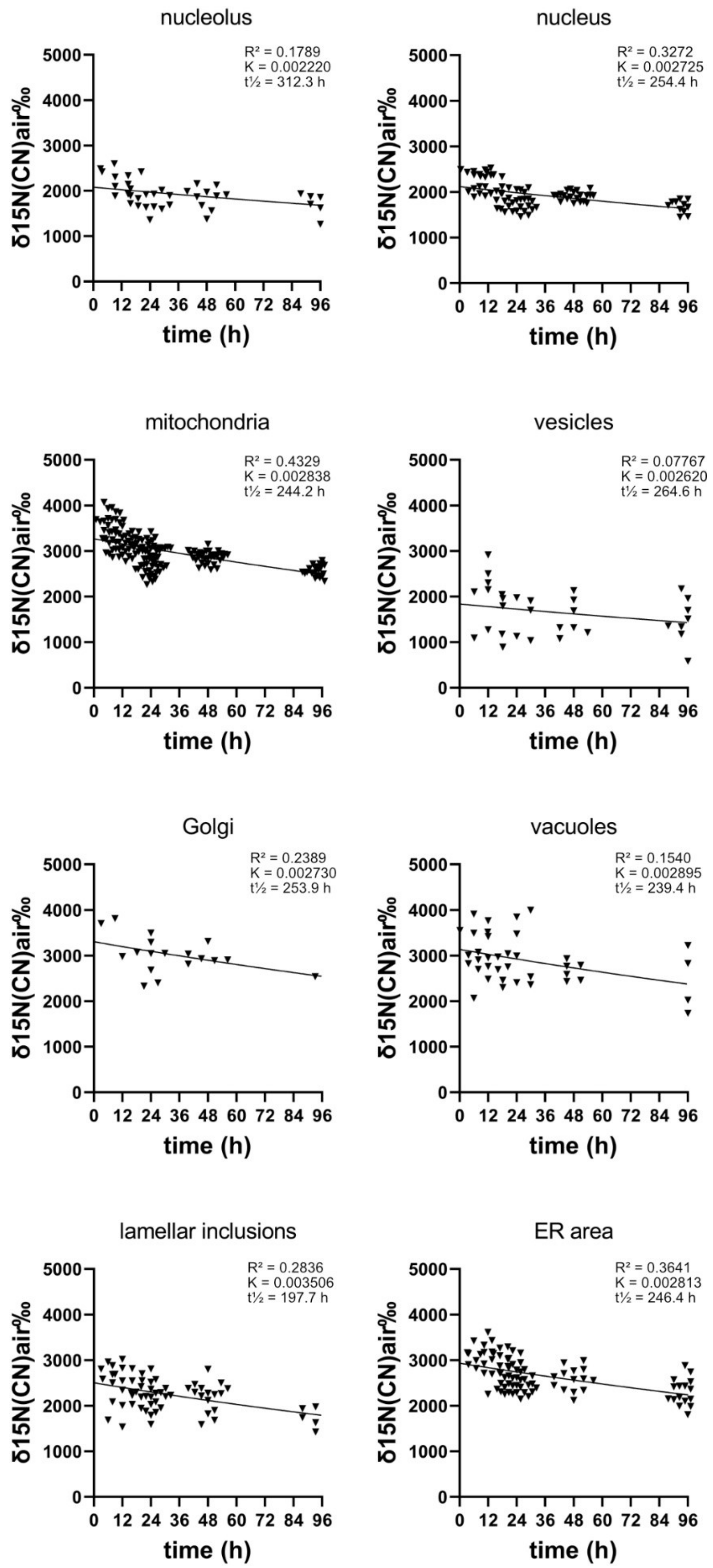

Figure S14: δ^{15}N over 96h chase time in ^{15}N-phenylalanine incubated cells. A one-phase decay curve is fitted to each dataset with GraphPad Prism 9.3.1 software; R^2, rate constant K and $t_{1/2}$ are given for each curve.

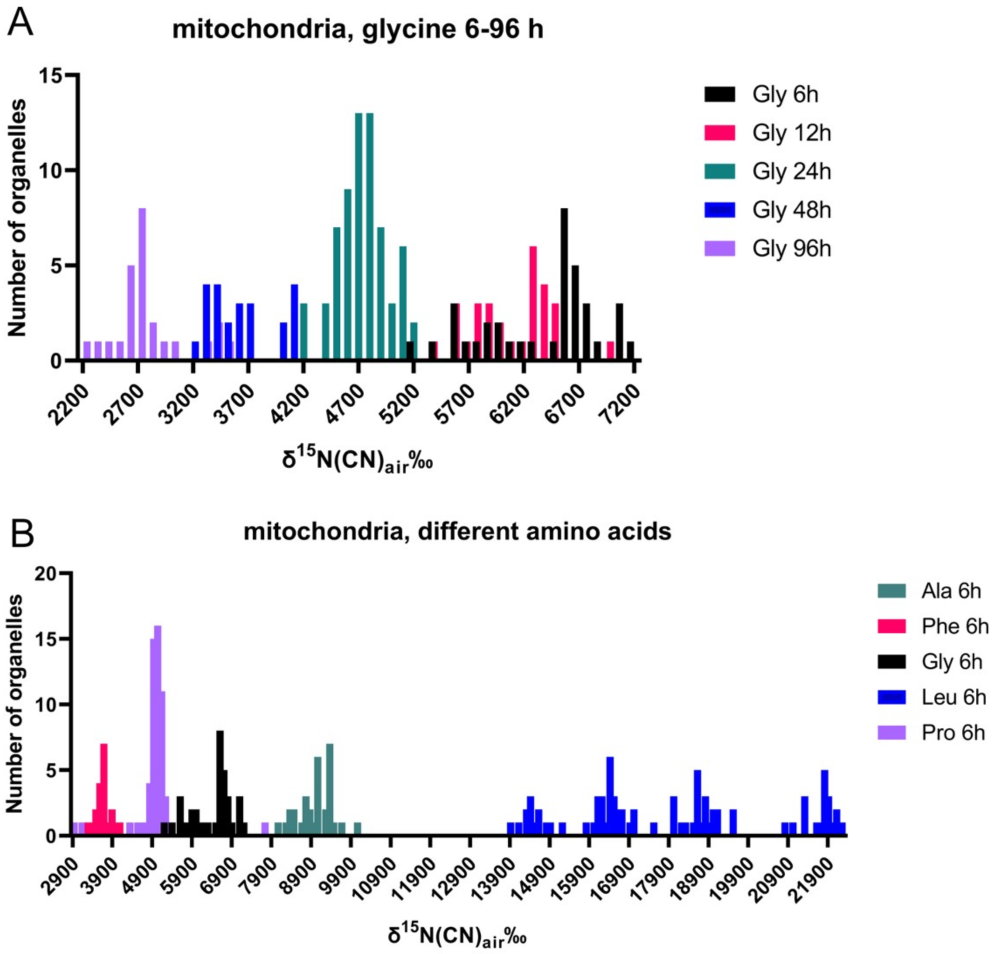

Figure 15: Histograms of the protein turnover of individual mitochondria from all the cells incubated with ^{15}N-glycine followed by different chase periods between 6h and 96h (A), and from all the cells incubated with different ^{15}N-amino acids followed by a 6h chase period (B). Ala: alanine, Phe: phenylalanine, Gly: glycine, Leu: leucine, Pro: proline.

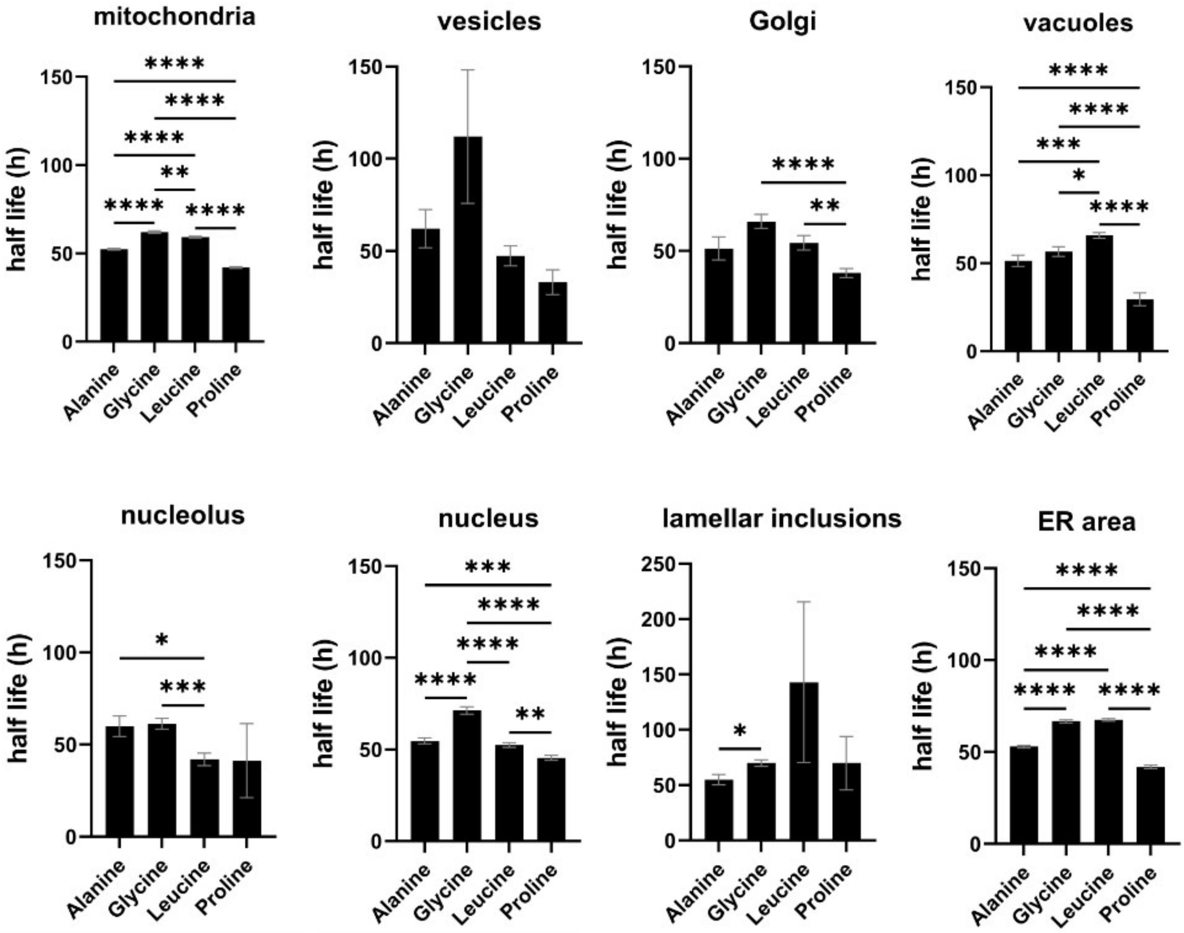

Figure S16: Protein half-lives of cellular organelles are significantly different corresponding to different incubated ^{15}N-amino acids. One-way ANOVA test (Brown Forsythe and Welch) was performed followed by Dunnett's multiple comparison. Significances from Dunnett's multiple comparisons for n<50 and from Games-Howell's multiple comparisons for n>50 (mitochondria, ER area, nucleus) are indicated as * p<.05, ** p<.01, *** p<.001, **** p<.0001.

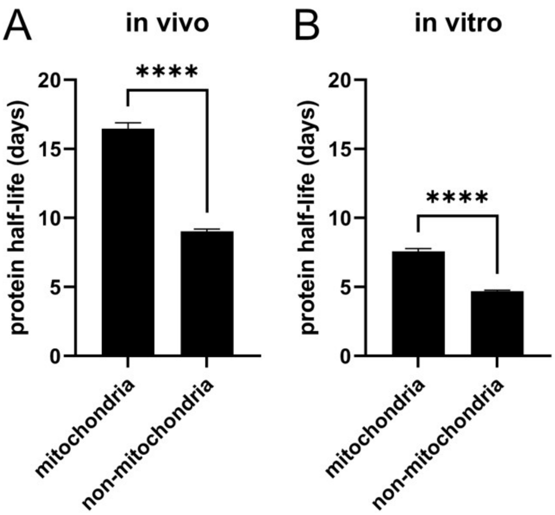

Figure S17: Differences between the lifetimes of proteins in mitochondria and elsewhere, from both *in vivo* data (mouse cortex)[2], and in vitro data (cultured neurons).[3] Both studies showed that mitochondria proteins display a far slower turnover than other cellular components.

Table S1: Summary of all significances of the $^{32}S/^{12}C^{12}C$ measurements after one-way ANOVA (Kruskal Wallis, Dunn's multiple comparisons test) was performed.

Dunn's multiple comparisons test	Mean rank diff.	Significant?	Summary	Adjusted P Value
nucelolus *vs* vesicles	1891	Yes	****	<0.0001
nucelolus *vs* mitochondria	-565.7	Yes	****	<0.0001
nucelolus *vs* nucleus	566	Yes	****	<0.0001
nucelolus *vs* Golgi	20.35	No	ns	>0.9999
nucelolus *vs* vacuoles	131.1	No	ns	>0.9999
nucelolus *vs* lamellar inclusion	1325	Yes	****	<0.0001
nucelolus *vs* cell cell connection	853.6	Yes	****	<0.0001
nucelolus *vs* centrosome	-1247	Yes	****	<0.0001
nucelolus *vs* ER area	493	Yes	****	<0.0001
vesicles *vs* mitochondria	-2457	Yes	****	<0.0001
vesicles *vs* nucleus	-1325	Yes	****	<0.0001
vesicles *vs* Golgi	-1870	Yes	****	<0.0001
vesicles *vs* vacuoles	-1760	Yes	****	<0.0001
vesicles *vs* lamellar inclusion	-566	Yes	****	<0.0001
vesicles *vs* cell cell connection	-1037	Yes	****	<0.0001
vesicles *vs* centrosome	-3138	Yes	****	<0.0001
vesicles *vs* ER area	-1398	Yes	****	<0.0001
mitochondria *vs* nucleus	1132	Yes	****	<0.0001
mitochondria *vs* Golgi	586	Yes	****	<0.0001
mitochondria *vs* vacuoles	696.8	Yes	****	<0.0001

mitochondria *vs* lamellar inclusion	1891	Yes	****	<0.0001
mitochondria *vs* cell cell connection	1419	Yes	****	<0.0001
mitochondria *vs* centrosome	-681.2	No	ns	0.0753
mitochondria *vs* ER area	1059	Yes	****	<0.0001
nucleus *vs* Golgi	-545.6	Yes	****	<0.0001
nucleus *vs* vacuoles	-434.9	Yes	****	<0.0001
nucleus *vs* lamellar inclusion	758.8	Yes	****	<0.0001
nucleus *vs* cell cell connection	287.6	No	ns	0.9234
nucleus *vs* centrosome	-1813	Yes	****	<0.0001
nucleus *vs* ER area	-73.04	No	ns	>0.9999
Golgi *vs* vacuoles	110.7	No	ns	>0.9999
Golgi *vs* lamellar inclusion	1304	Yes	****	<0.0001
Golgi *vs* cell cell connection	833.2	Yes	****	<0.0001
Golgi *vs* centrosome	-1267	Yes	****	<0.0001
Golgi *vs* ER area	472.6	Yes	***	0.0006
vacuoles *vs* lamellar inclusion	1194	Yes	****	<0.0001
vacuoles *vs* cell cell connection	722.5	Yes	****	<0.0001
vacuoles *vs* centrosome	-1378	Yes	****	<0.0001
vacuoles *vs* ER area	361.9	Yes	****	<0.0001
lamellar inclusion *vs* cell cell connection	-471.3	Yes	*	0.019
lamellar inclusion *vs* centrosome	-2572	Yes	****	<0.0001
lamellar inclusion *vs* ER area	-831.9	Yes	****	<0.0001
cell cell connection *vs* centrosome	-2101	Yes	****	<0.0001
cell cell connection *vs* ER area	-360.6	No	ns	0.1306
centrosome *vs* ER area	1740	Yes	****	<0.0001

Table S2: Summary of all significances of the $^{31}P/^{12}C^{12}C$ measurements after one-way ANOVA (Kruskal Wallis, Dunn's multiple comparisons test) was performed.

**Dunn's multiple comparisons test**	**Mean rank diff.**	**Significant?**	**Summary**	**Adjusted P Value**
nucelolus *vs* vesicles	1277	Yes	****	<0.0001
nucelolus *vs* mitochondria	1310	Yes	****	<0.0001
nucelolus *vs* nucleus	809.4	Yes	****	<0.0001
nucelolus *vs* Golgi	1675	Yes	****	<0.0001
nucelolus *vs* vacuoles	2217	Yes	****	<0.0001
nucelolus *vs* lamellar inclusion	738.7	Yes	****	<0.0001
nucelolus *vs* cell cell connection	1682	Yes	****	<0.0001
nucelolus *vs* centrosome	2977	Yes	****	<0.0001
nucelolus *vs* ER area	1209	Yes	****	<0.0001
vesicles *vs*	32.99	No	ns	>0.9999

mitochondria				
vesicles *vs* nucleus	-467.4	Yes	****	<0.0001
vesicles *vs* Golgi	398	No	ns	0.0982
vesicles *vs* vacuoles	940.2	Yes	****	<0.0001
vesicles *vs* lamellar inclusion	-538.1	Yes	****	<0.0001
vesicles *vs* cell cell connection	404.8	No	ns	0.1777
vesicles *vs* centrosome	1700	Yes	****	<0.0001
vesicles *vs* ER area	-67.96	No	ns	>0.9999
mitochondria *vs* nucleus	-500.4	Yes	****	<0.0001
mitochondria *vs* Golgi	365	Yes	*	0.0291
mitochondria *vs* vacuoles	907.2	Yes	****	<0.0001
mitochondria *vs* lamellar inclusion	-571.1	Yes	****	<0.0001
mitochondria *vs* cell cell connection	371.8	No	ns	0.0846
mitochondria *vs* centrosome	1667	Yes	****	<0.0001
mitochondria *vs* ER area	-101	No	ns	>0.9999
nucleus *vs* Golgi	865.3	Yes	****	<0.0001
nucleus *vs* vacuoles	1408	Yes	****	<0.0001
nucleus *vs* lamellar inclusion	-70.75	No	ns	>0.9999
nucleus *vs* cell cell connection	872.2	Yes	****	<0.0001
nucleus *vs* centrosome	2168	Yes	****	<0.0001
nucleus *vs* ER area	399.4	Yes	****	<0.0001
Golgi *vs* vacuoles	542.3	Yes	***	0.0002
Golgi *vs* lamellar inclusion	-936.1	Yes	****	<0.0001
Golgi *vs* cell cell connection	6.848	No	ns	>0.9999
Golgi *vs* centrosome	1302	Yes	****	<0.0001
Golgi *vs* ER area	-465.9	Yes	***	0.0008
vacuoles *vs* lamellar inclusion	-1478	Yes	****	<0.0001
vacuoles *vs* cell cell connection	-535.4	Yes	**	0.0016
vacuoles *vs* centrosome	760.2	Yes	*	0.0283
vacuoles *vs* ER area	-1008	Yes	****	<0.0001
lamellar inclusion *vs* cell cell connection	942.9	Yes	****	<0.0001
lamellar inclusion *vs* centrosome	2239	Yes	****	<0.0001
lamellar inclusion *vs* ER area	470.2	Yes	****	<0.0001
cell cell connection *vs* centrosome	1296	Yes	****	<0.0001
cell cell connection *vs* ER area	-472.8	Yes	**	0.0043
centrosome *vs* ER area	-1768	Yes	****	<0.0001

Table S3: Summary of all significances of the $^{12}C^{14}N/^{12}C^{12}C$ measurements after one-way ANOVA (Kruskal Wallis, Dunn’s multiple comparisons test) was performed.

**Dunn's multiple comparisons test**	**Mean rank diff.**	**Significant?**	**Summary**	**Adjusted P Value**
nucelolus *vs* vesicles	3104	Yes	****	<0.0001
nucelolus *vs* mitochondria	979.6	Yes	****	<0.0001
nucelolus *vs* nucleus	885	Yes	****	<0.0001
nucelolus *vs* Golgi	1668	Yes	****	<0.0001
nucelolus *vs* vacuoles	2387	Yes	****	<0.0001
nucelolus *vs* lamellar inclusion	1929	Yes	****	<0.0001
nucelolus *vs* cell cell connection	2614	Yes	****	<0.0001
nucelolus *vs* centrosome	390.9	No	ns	>0.9999
nucelolus *vs* ER area	1691	Yes	****	<0.0001
vesicles vs mitochondria	-2124	Yes	****	<0.0001
vesicles *vs* nucleus	-2219	Yes	****	<0.0001
vesicles *vs* Golgi	-1436	Yes	****	<0.0001
vesicles *vs* vacuoles	-716.6	Yes	****	<0.0001
vesicles *vs* lamellar inclusion	-1175	Yes	****	<0.0001
vesicles *vs* cell cell connection	-490.3	Yes	*	0.0212
vesicles *vs* centrosome	-2713	Yes	****	<0.0001
vesicles *vs* ER area	-1413	Yes	****	<0.0001
mitochondria *vs* nucleus	-94.61	No	ns	>0.9999
mitochondria *vs* Golgi	688.8	Yes	****	<0.0001
mitochondria *vs* vacuoles	1408	Yes	****	<0.0001
mitochondria *vs* lamellar inclusion	949.5	Yes	****	<0.0001
mitochondria *vs* cell cell connection	1634	Yes	****	<0.0001
mitochondria *vs* centrosome	-588.7	No	ns	0.3017
mitochondria *vs* ER area	711.1	Yes	****	<0.0001
nucleus *vs* Golgi	783.4	Yes	****	<0.0001
nucleus *vs* vacuoles	1502	Yes	****	<0.0001
nucleus *vs* lamellar inclusion	1044	Yes	****	<0.0001
nucleus *vs* cell cell connection	1729	Yes	****	<0.0001
nucleus *vs* centrosome	-494.1	No	ns	>0.9999
nucleus *vs* ER area	805.7	Yes	****	<0.0001
Golgi *vs* vacuoles	719	Yes	****	<0.0001
Golgi *vs* lamellar inclusion	260.7	No	ns	>0.9999
Golgi *vs* cell cell connection	945.3	Yes	****	<0.0001
Golgi *vs* centrosome	-1278	Yes	****	<0.0001
Golgi *vs* ER area	22.29	No	ns	>0.9999
vacuoles *vs* lamellar inclusion	-458.3	Yes	****	<0.0001
vacuoles *vs* cell cell connection	226.3	No	ns	>0.9999
vacuoles *vs* centrosome	-1996	Yes	****	<0.0001

vacuoles *vs* ER area	-696.7	Yes	****	<0.0001
lamellar inclusion *vs* cell cell connection	684.6	Yes	****	<0.0001
lamellar inclusion *vs* centrosome	-1538	Yes	****	<0.0001
lamellar inclusion *vs* ER area	-238.4	No	ns	0.1123
cell cell connection *vs* centrosome	-2223	Yes	****	<0.0001
cell cell connection *vs* ER area	-923	Yes	****	<0.0001
centrosome *vs* ER area	1300	Yes	****	<0.0001

Reference

1 Bell S, Hettige NC, Silveira H, Peng H, Wu H, Jefri M, Antonyan L, Zhang Y, Zhang X, Ernst C. Differentiation of Human Induced Pluripotent Stem Cells (iPSCs) into an Effective Model of Forebrain Neural Progenitor Cells and Mature Neurons. *Bio Protoc.* Mar 5;9(5):e3188 (2019).

2 Fornasiero E.F., Mandad S., Wildhagen H. et al. Precisely measured protein lifetimes in the mouse brain reveal differences across tissues and subcellular fractions. *Nat Commun* **9**, 4230 (2018).

3 Cohen LD, Zuchman R, Sorokina O, et al. Metabolic turnover of synaptic proteins: kinetics, interdependencies and implications for synaptic maintenance. *PLoS One*. **2**, 8, 5, e63191 (2013).
